# Integrated epigenetic biomarkers in circulating cell-free DNA as a robust classifier for pancreatic cancer

**DOI:** 10.1186/s13148-020-00898-2

**Published:** 2020-07-23

**Authors:** Feng Cao, Ailin Wei, Xinlei Hu, Yijing He, Jun Zhang, Lin Xia, Kailing Tu, Jue Yuan, Ziheng Guo, Hongying Liu, Dan Xie, Ang Li

**Affiliations:** 1 Frontier Science Center for Disease Molecular Network, State Key Laboratory of Biotherapy, West China Hospital, Sichuan University, Chengdu, 610041 Sichuan Province China; 2 Department of Pancreatic Surgery, West China Hospital, Sichuan University, Chengdu, 610041 Sichuan Province China; 3 Key Laboratory of Transplant Engineering and Immunology, Regenerative Medicine Research Center, West China Hospital, Sichuan University, Chengdu, 610041 Sichuan Province China

**Keywords:** Cell-free 5mC sequencing, Cell-free 5hmC sequencing, Liquid biopsy, Pancreatic cancer, Cancer diagnosis

## Abstract

**Background:**

The high lethal rate of pancreatic cancer is partly due to a lack of efficient biomarkers for screening and early diagnosis. We attempted to develop effective and noninvasive methods using 5-methylcytosine (5mC) and 5-hydroxymethylcytosine (5hmC) markers from circulating cell-free DNA (cfDNA) for the detection of pancreatic ductal adenocarcinoma (PDAC).

**Results:**

A 24-feature 5mC model that can accurately discriminate PDAC from healthy controls (area under the curve (AUC) = 0.977, sensitivity = 0.824, specificity = 1) and a 5hmC prediction model with 27 features demonstrated excellent detection power in two distinct validation sets (AUC = 0.992 and 0.960, sensitivity = 0.786 and 0.857, specificity = 1 and 0.993). The 51-feature model combining 5mC and 5hmC markers outperformed both of the individual models, with an AUC of 0.997 (sensitivity = 0.938, specificity = 0.955) and particularly an improvement in the prediction sensitivity of PDAC. In addition, the weighted diagnosis score (wd-score) calculated with the 5hmC model can distinguish stage I patients from stage II–IV patients.

**Conclusions:**

Both 5mC and 5hmC biomarkers in cfDNA are effective in PDAC detection, and the 5mC-5hmC integrated model significantly improve the detection sensitivity.

**Graphical abstract:**

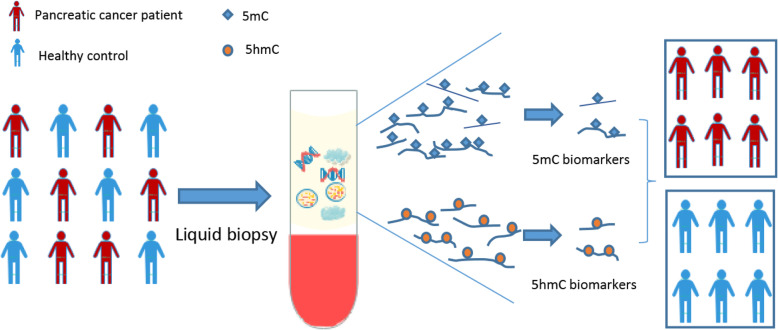

## Background

Pancreatic cancer is a highly lethal disease, as most patients are asymptomatic until they are in an advanced stage [1]. Pancreatic ductal adenocarcinoma (PDAC) patients benefit most from early diagnosis and surgery. Once distant metastasis occurs, any systemic therapy is rarely curative. Therefore, a major goal in PDAC research is the detection of cancer when effective surgery can be performed.

Liquid biopsies, a well-known noninvasive method, have aroused public attention as diagnostic materials for cancer, particularly circulating tumor DNA (ctDNA) in plasma. Taking advantage of technical advances, both genetic and epigenetic aberrations of cell-free DNA (cfDNA) can be detected [2] and have shown promising performance in clinical practice, including diagnosis [3–8], prognosis [9–12], and drug resistance [13, 14].

Epigenetic mechanisms play critical roles in individual development and tissue-specific gene expression, while their dysregulation frequently occurs in human diseases, notably cancer [15, 16]. Global changes in epigenetic modifications, such as 5-methylcytosine (5mC) and 5-hydroxymethylcytosine (5hmC), are hallmarks of cancer [17, 18]. It was suggested that epigenetic modifiers, which are regulated by epigenetic modulators serving to transduce signals from environmental factors, modified tumor progenitor genes to influence gene expression, which was believed to be the earliest stage of carcinogenesis [19].

Considering the low mutation frequency of tumor-related somatic mutations and the limited detection sensitivity, 5mC and 5hmC in cfDNA could serve as parallel or even more valuable biomarkers [20]. Recent technological improvements in 5mC and 5hmC detection from cfDNA, including the cell-free methylated DNA immunoprecipitation and high-throughput sequencing (cfMeDIP-seq) method [21, 22] and cell-free 5hmC sequencing methods reported recently [20, 23], offer substantial advantages over previous ctDNA 5mC and 5hmC detection methods. Therefore, 5mC detection and 5hmC characterization in cfDNA are anticipated to be robust and cost-effective methods for clinical application in cancer diagnosis and therapy [24].

We employed cfMeDIP-seq and cell-free 5hmC sequencing to explore the application potential of epigenetic markers in noninvasive diagnosis and attempted to test whether the combination of the two types of epigenetic markers could improve the diagnostic power. We developed three prediction models for PDAC detection, including a 5mC model with 24 features, a 5hmC model with 27 features, and an integrated model using the 51 features identified. In addition, we investigated the genomic distribution of 5mC and 5hmC as well as the modification level changes at H3K36me3, H3K27ac, H3K4me3, H3K4me1, and H3K27me3.

## Results

### Characterization of the cfMeDIP-seq data and cfDNA 5hmC sequencing data

We recruited 136 healthy individuals and 72 PDAC patients of Chinese descent in our study (Table 1), of which 61 PDAC and 86 healthy controls had paired cell-free 5mC and 5hmC data. CfMeDIP-seq data from 97 healthy controls and 67 PDAC samples were qualified for methylome analysis. The spike-in control with sequencing adaptors demonstrated specific 5mC enrichment (Supplementary Figure 1A). The median final unique non-duplicate mapping rate of the cfMeDIP-seq libraries was 0.8, and the median total reads was ~ 17.4 M (Supplementary Table 1).

**Table 1:** Demographical and clinicopathological characteristics of all the participants

Parameters	PDAC group (*N* = 72)	Healthy control group (*N* = 136)	*P* value
Age, average ± standard error	59.54 ± 1.19	58.23 ± 0.75	0.379
Gender, *n* (%)			
Male	41(57%)	48(35%)	0.003
BMI, average ± standard error	22.03 ± 0.37	23.81 ± 0.30	< 0.0001
Smoking history, *n* (%)	22(31%)	19(14%)	0.004
Alcohol history, *n* (%)	25(35%)	19(14%)	< 0.0001
Chronic disease, *n* (%)			
Hypertension	9(13%)	37(27%)	0.015
Type II diabetes	13(18%)	15(11%)	0.158
CA199, average ± standard error	357.29 ± 42.28	/	
Jaundice	21(29%)	0	< 0.0001
Tumor size, average ± standard error	3.79 ± 0.19	/	
Primary cancer site, *n* (%)		/	
Uncinate process	13(18%)		
Head	29(40%)		
Body	17(24%)		
Tail	13(18%)		
Surgery, *n* (%)		/	
Pancreaticoduodenectomy	26(36%)		
Distal pancreatectomy	11(15%)		
Palliative intervention techniques	35(49%)		
AJCC staging, *n* (%)		/	
I	8(11%)		
II	28(39%)		
III	18(25%)		
IV	18(25%)		

*BMI* body mass index, *CA199* carbohydrate antigen199, *AJCC* American Joint Committee on Cancer

*Note*: *P* values were calculated using chi-squared test

Considering that cfDNA 5hmC signatures in PDAC also deserve deep inquiry, 5hmC profiling data from 136 healthy controls and 67 PDAC samples were generated. The count of reads mapping to the spike-in control demonstrated highly specific enrichment of 5hmC fragments (Supplementary Figure 1B). The final 5hmC libraries were highly complex (a median unique non-duplicate rate of 0.83) with a relatively low sequencing depth (median 20.8 M reads) (Supplementary Table 2).

### Genome-wide profiling of 5mC and 5hmC in cfDNA

To explore the distribution patterns of methylation in cfDNA across the genome, we defined the 201 bp fixed-width peaks called by MACS2 as 5mC-enriched regions. Comparing the peak number between PDAC samples and healthy controls, no significant difference was observed, though the median peak number of PDAC was greater than that of the control group (Supplementary Figure 2A). However, the total number of 5hmC peaks captured from the PDAC samples was significantly less than that captured from the healthy controls (*P* value = 1.43E−05) (Supplementary Figure 2B). Considering that the peak number could not fully represent the global modification level, we inspected the 5mC change with the Integrative Genomics Viewer (IGV) [25, 26]. No significant global 5mC depletion was observed in the PDAC samples, only demethylation within relatively small ranges. In contrast, global hypermethylation regions were observed (Supplementary Figure 2C). Next, we checked the global 5hmC level change by IGV. The result showed global 5hmC loss in the PDAC samples (Supplementary Figure 2D).

By locating these peaks to distinct genome elements, we observed that the 5mC-enriched regions were significantly enriched in CDS, exons, 5′UTRs, 3′UTRs, and promoters, with no significant change in the PDAC samples compared to the controls, while depleted in introns and intergenic regions (Supplementary Figure 3A, B).

Analysis of the 5hmC peaks showed that the 5hmC-enriched regions are highly enriched in CDS, 5′UTRs, exons, 3′UTRs, and promoters, while depleted in introns and intergenic regions. The PDAC samples showed a significantly higher odds ratio in 5′UTRs, 3′UTRs, and exons, while no significant difference was observed within promoters and CDS compared to the controls (Supplementary Figure 3C, D).

### Comparing the distributions of the 5mC and 5hmC peaks

**Fig. 1:**
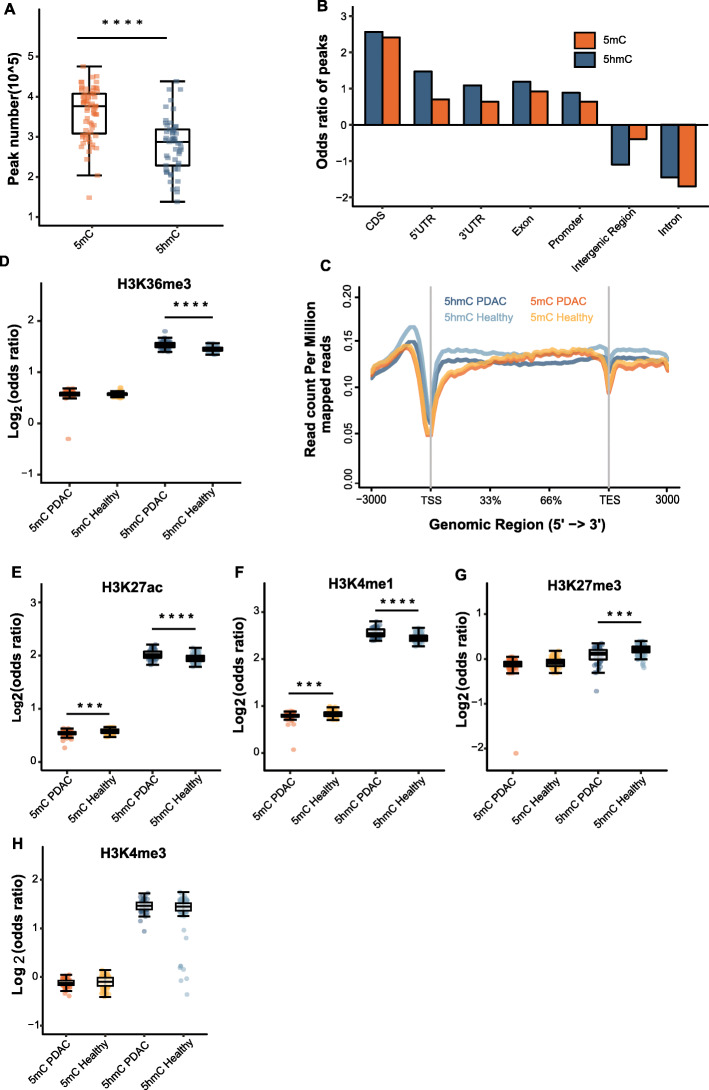
Comparison of genome-wide distribution between 5mC and 5hmC. **a** Boxplot of the total number of the 5mC peaks and 5hmC peaks in PDAC samples. **b** Enrichment analysis of the 5mC and 5hmC peaks overlapping with distinct genomic elements. **c** Metagene profiles of the regions from TSS to TES with the flanking 3 kb. Boxplots of Log_2_ (odds ratio) of peaks overlapping with H3K36me3 (**d**), H3K27ac (**e**), H3K4me1 (**f**), H3K27me3 (**g**), and H3K4me3 (**h**). *P < 0.05, **P < 0.01, ***P < 0.001, ****P < 1e−5, Wilcoxon test. PDAC, pancreatic ductal adenocarcinoma; TSS, transcription start sites; TES, transcription end sites

First, we compared the peak numbers of the 5mC and 5hmC profiles. The total number of 5mC peaks (*n* = 772,365) was approximately 2-folds greater than that of the 5hmC peaks (Fig. 1a). However, only 16.7% overlapped with 5hmC peak sites. Moreover, 30% of 5hmC sites also had 5mC modifications (Supplementary Figure 4A). There were 17,340 genes carrying both 5mC and 5hmC modifications, of which 3303 genes (~ 16% of the total genes detected with 5mC and/or 5hmC modification) had 5mC modifications only, and 124 genes were specifically modified with 5hmC (Supplementary Figure 4B). The results suggested that over 80% of 5mC peaks occurred at sites distinct to 5hmC, while genes frequently bear the two types of modifications.

Next, we investigated the functions of the genes with specific 5mC or specific 5hmC modifications. The 5mC-specific genes were mainly enriched in the olfactory signaling pathway, keratinization, and GPCR ligand binding, et al. (Supplementary Figure 5A), while the 5hmC-specific genes were enriched in oxidative stress–induced senescence (Supplementary Figure 5B). Supplementary Figure 6 demonstrates examples of 5mC-specific genes and 5hmC-specific genes.

Comparing the genome distribution patterns of 5hmC and 5mC, we observed that 5hmC exhibited significantly higher enrichment in CDS, 5′UTRs, 3′UTRs, exons, and promoters. Both 5mC and 5hmC demonstrated depletion in introns and intergenic regions. 5hmC depletion within intergenic regions in PDAC samples showed a much greater extent than 5mC, while the opposite trend was observed within introns (Fig. 1b). Reduced 5hmC modifications within gene bodies in PDAC were observed by metagene analysis (Fig. 1c). The changes of 5mC modifications within gene bodies were not as significant as that of the 5hmC modifications in the regions in the PDAC samples (Fig. 1c).

Given that histone modifications have a biological relationship with DNA methylation [27], we investigated the overlap of 5mC and 5hmC modification peaks in five types of histone modifications, including H3K36me3, H3K27ac, H3K4me1, H3K4me3, and H3K27me3. The intersection of 5hmC profiling data with the histone map of the PANC-1 cell line from the ENCODE Project exhibited increased fragments per kilobase of gene per million mapped reads (FPKM) in H3K36me3 (*P* value = 7.25E−12), H3K27ac (*P* value = 2.55E−06), and H3K4me1 (*P* value = 1.79E−09) in the PDAC cohorts relative to the healthy cohorts (Fig. 1d–f). In contrast, 5mC modifications located in H3K4me1 (*P* value = 2.73E−05) and H3K27ac (*P* value = 1.59E−05) peaks decreased in the PDAC samples compared to those in the healthy control samples (Fig. 1d, e). Given that H3K36me3, H3K27ac, and H3K4me1 are marks of active regulatory elements [28], increased 5hmC levels and reduced 5mC levels in these regions suggested the active transcription of genes regulated by the elements. For the repressive histone modification H3K27me3, 5hmC level significantly decreased in PDAC samples (*P* value = 5.80E−05), while 5mC level changes were not statistically significant (Fig. 1g). The density of both 5hmC and 5mC in H3K4me3 sites presented no significant difference (Fig. 1h).

### Prediction of PDAC by using 5mC biomarkers in cfDNA

**Fig. 2:**
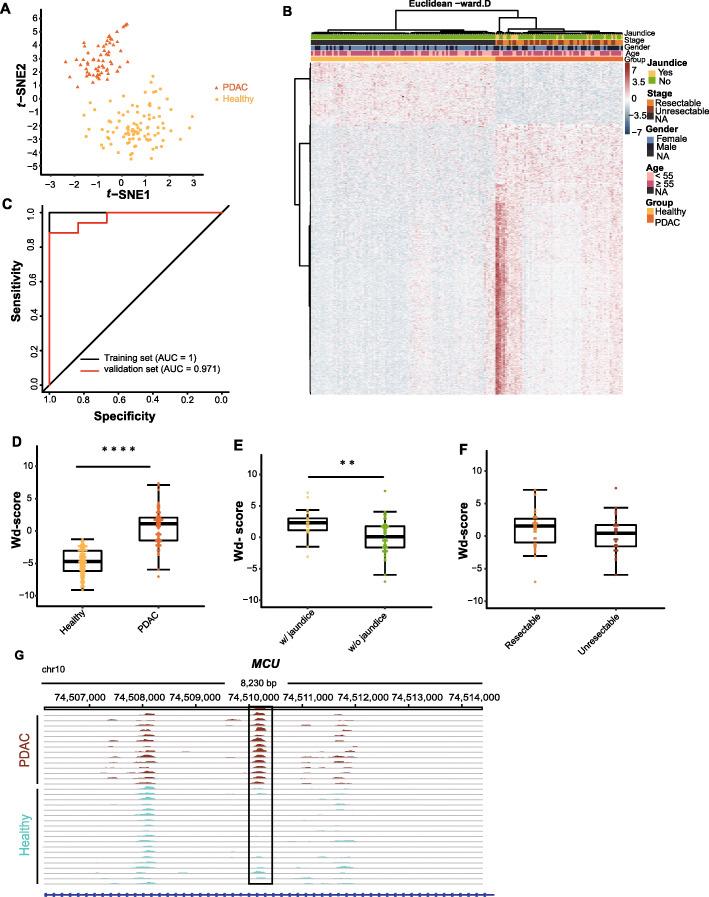
Cell-free 5mC for detection of PDAC. T-SNE plot (**a**) and heatmap (**b**) of 5mC FPKM from the training set and the validation set based on the 688 differentially methylated peaks (DMPs). Hierarchical clustering was performed across peaks and samples. **c** Performance of the 5mC model in the training set and the validation set. **d** Boxplot of the wd-scores calculating with the 5mC model for the PDAC samples and the healthy controls. **e** Boxplot of the wd-scores of the PDAC patients with jaundice and those without jaundice. **f** Boxplot of the wd-scores of the resectable PDAC patients and the unresectable patients. **g** Genome Browser view of the 5hmC peaks in *MCU* gene in chromosome 10 shows a marker locating within the gene (boxed region: chr10: 74510104-74510305). *P < 0.05, **P < 0.01, ***P < 0.001, ****P < 1e−5, Wilcoxon test. PDAC, pancreatic ductal adenocarcinoma; AUC, area under the curve; wd-score, weighted diagnosis score

The fragments per kilobase of gene per million mapped reads (FPKM) of each peak were calculated in every single sample regarding its 5mC modification level. T-distributed stochastic neighbor embedding (*t-*SNE) analysis with the consensus peak sets showed a slight batch effect, which was eliminated by Combat (sva package) (Supplementary Figure 7). Based on the consensus peak sets and datasets without batch effects, we identified 688 differentially methylated peaks (DMPs) in the 67 PDAC samples and 97 healthy controls analyzed (Student’s *t* test, *P* value < 0.01, |log fold change (FC)| > 0.8), including 560 hypermethylated peaks in the cases and 128 hypermethylated peaks in the controls (Supplementary Table 3). The healthy controls and PDAC samples could be classified by hierarchical clustering and *t-*SNE using the 688 DMPs identified, and no association with resectable stage and jaundice was observed (Fig. 2a, b). Utilizing the above methods and criteria to identify DMPs, the elastic-net method was used for model construction with the set of 164 samples, partitioned randomly into a training set consisting of 50 PDAC samples and 67 healthy controls and a validation set comprising 17 PDAC samples and 30 healthy controls (Supplementary Figure 8). Finally, 24 biomarkers (alpha = 0.2) (Supplementary Table 4) that appeared in at least 3 training subsets were selected for the final model, achieving a sensitivity of 100% and a specificity of 100% (area under the curve (AUC) = 1) (Fig. 2c) in the training set. To assess the prediction ability of the classifier, 17 PDAC samples and 30 healthy controls were used for validation, exhibiting a sensitivity of 82.4% and a specificity of 100% (AUC = 0.971) (Fig. 2c).

The differences in the weighted diagnosis scores (wd-scores) calculated with the model (Supplementary Table 5) between the PDAC group and the healthy group were statistically significant (*P* value = 1.15E−22) (Fig. 2d). Unexpectedly, the wd-score divergence between PDAC patients with jaundice and patients without jaundice was statistically significant (*P* value = 4.64E−03) (Fig. 2e). However, no significant difference in the wd-score was observed between the resectable PDAC group and unresectable group, despite the former obtaining a higher median wd-score (Fig. 2f). A portion of the markers identified in this model were mapped to tumor-related genes, such as *MCU*, *KRT80*, and *MGAT5*. Figure 2g exhibited the IGV plot of the marker locating in *MCU* gene. The PDAC samples showed increased 5mC level of the marker when compared with the healthy controls.

### Prediction of PDAC by using 5hmC biomarkers in cfDNA

**Fig. 3:**
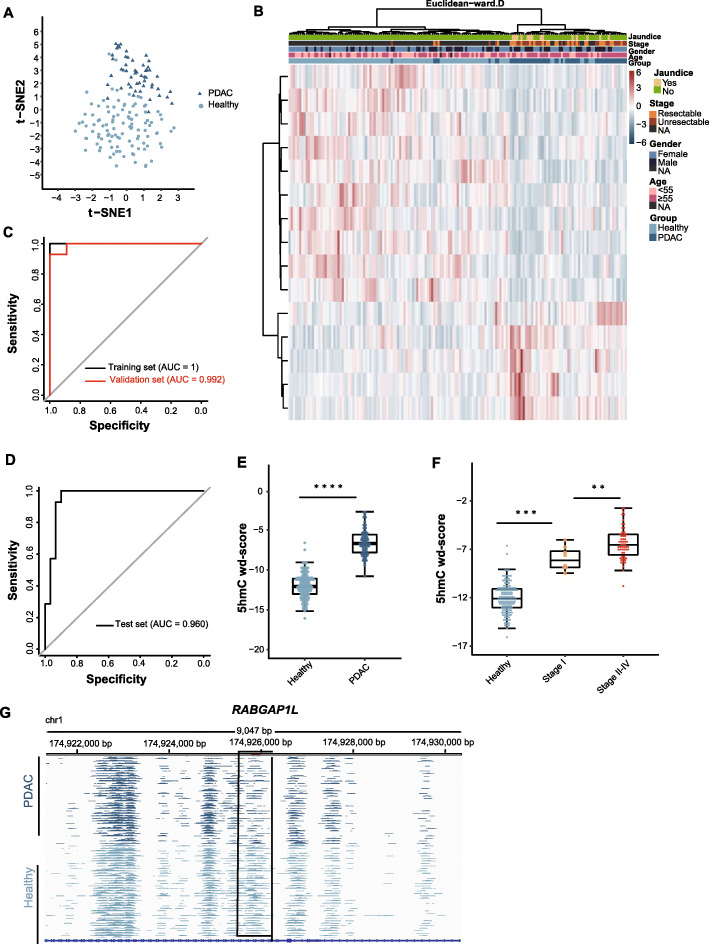
Cell-free 5hmC for detection of PDAC. T-SNE plot (**a**) and heatmap (**b**) of 5hmC FPKM from the training set and the validation set based on the 15 differentially hydroxymethylated peaks (DhMPs). Hierarchical clustering was performed across peaks and samples. **c** Performance of the 5hmC model in the training set and the validation set. **d** Performance of the 5hmC model in the test data set. **e** Boxplot of the wd-scores calculating with 5hmC model for PDAC samples and healthy controls. **f** Boxplot of the wd-scores calculating with 5hmC model for healthy controls, stage I PDAC samples and stage II–IV PDAC samples. *P < 0.05, **P < 0.01, ***P < 0.001, ****P < 1e−5, ANOVA. **g** Genome Browser view of the 5hmC peaks in *RABGAP1L* gene in chromosome 6 shows a marker locating within the gene (boxed region: chr1: 174799728-174799929). PDAC, pancreatic ductal adenocarcinoma; AUC, area under the curve; wd-score, weighted diagnosis score

No significant batch effects were observed among all cfDNA 5hmC sequencing data (Supplementary Figure 9). We performed Student’s *t* test to determine the optimal parameter for differentially hydroxymethylated peak (DhMP) identification, and 15 DhMP features were characterized within 53 PDAC samples and 106 healthy controls (Student’s *t* test, *P* value < 0.001, |log2FC| > = 0.8), comprising 6 hyperhydroxymethylated peaks and 9 hypohydroxymethylated peaks in the cases (Supplementary Table 6). *T-*SNE analysis and hierarchical clustering of 53 PDAC samples and 106 healthy control samples with the 15 DhMPs can separate PDAC from healthy controls (Fig. 3a, b). To explore the potential diagnostic value of 5hmC cfDNA and search for more effective biomarkers, the 159 total samples were randomly divided into a training set and a validation set comprising 75% and 25% of the data, respectively, with the elastic-net regularized regression method employed (Supplementary Figure 10). This approach suggested a set of 27 features of 5hmC profiles (Supplementary Table 7), resulting a discrimination model demonstrating high accuracy in both the training set (sensitivity = 97.4%, specificity = 100%, AUC = 1) and the validation set (sensitivity = 78.6%, specificity = 100%, and AUC = 0.992) (Fig. 3c).

Furthermore, 14 PDAC samples and 30 healthy controls were used to validate the model’s performance, achieving a sensitivity of 85.7% and a specificity of 93.3% (AUC = 0.960) (Fig. 3d). The wd-score showed an upward trend from healthy controls to PDAC patients, with significantly higher wd-scores in the PDAC samples (*P* value = 5.98E−30) (Fig. 3e). Interestingly, the wd-score between early-stage (American Joint Committee on Cancer (AJCC) stage I) and late-stage (AJCC stages II, III, and IV) patients demonstrated statistically significant disparity (*P* value = 7.00E−03), which suggested the capacity of the model to discriminate early- from late-stage patients (Fig. 3f). No significant difference between the resectable and unresectable PDAC groups was observed (Supplementary Figure 11A), while a significant difference in the wd-score was observed between the groups with and without jaundice (4.60E−02) (Supplementary Figure 11B). Figure 3g presents the 5hmC biomarker located at the *RABGAP1L* gene, which plays a key role in tumorigenesis. The 5hmC modification level of the DhMP at the *RABGAP1L* gene reduced in PDAC patients versus normal controls.

### Integrated model of 5mC-5hmC biomarkers

**Fig. 4:**
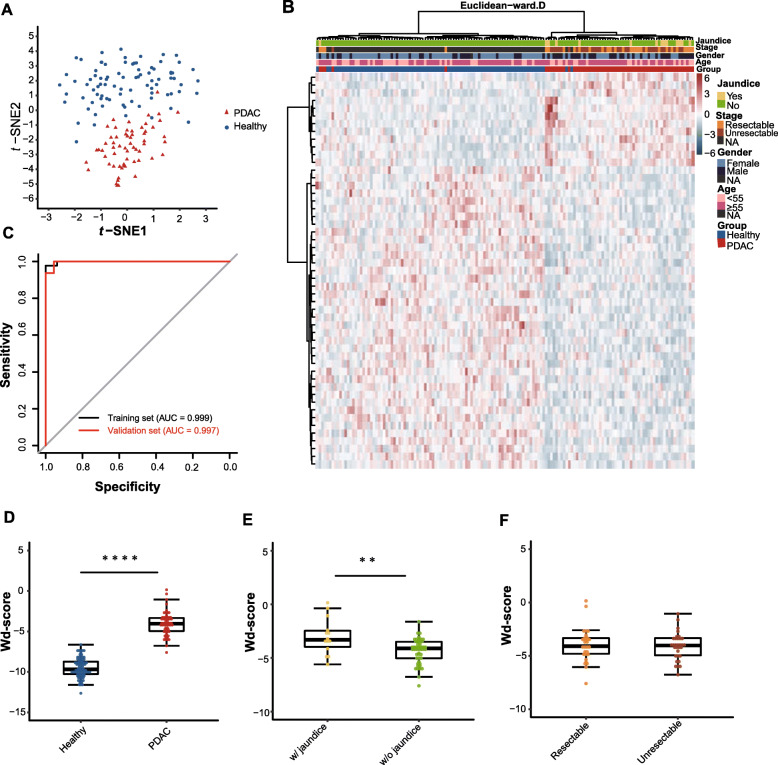
Performance of the 5mC-5hmC integrated model for PDAC detection. T-SNE plot (**a**) and heatmap (**b**) of FPKM from the paired 5mC and 5hmc data, based on the 51 features comprising the twenty-four 5mC biomarkers and the twenty-seven 5hmC biomarkers. Hierarchical clustering was performed across peaks and samples. **c** Performance of the 5mC-5hmC integrated model in the training set and the validation set. **d** Boxplot of wd-score deriving from the integrated model for the PDAC samples and the healthy controls. **e** Boxplot of the wd-scores from the integrated model for PDAC patients with jaundice and those without jaundice. **f** Boxplot of the wd-scores from the integrated model for the resectable PDAC patients and the unresectable PDAC patients. *P < 0.05, **P < 0.01, ***P < 0.001, ****P < 1e−5, Wilcoxon test. PDAC, pancreatic ductal adenocarcinoma; AUC, area under the curve; wd-score, weighted diagnosis score

Because 5mC and 5hmC depict different aspects of the epigenome, we envisioned that conjoint analysis would increase diagnostic power. To test this hypothesis, the 5hmC features (*n* = 27) and 5mC features (*n* = 24) identified were combined to construct a classification model with paired 5mC and 5hmC datasets from PDAC samples (*n* = 61) and healthy samples (*n* = 86). Both hierarchical clustering and *t-*SNE analysis indicated that the 51 features can discriminate PDAC from healthy samples (Fig. 4a, b). With the elastic-net modeling method, sensitivities of 97.8% and 93.8% and specificities of 100% and 95.5% (AUC = 0.999 and 0.997) (Fig. 4c) were achieved in the training and validation datasets respectively, exhibiting better performance than the models using the 5hmC or 5mC biomarkers alone. We also applied this model to calculate the wd-score for every single sample. The disparity of the wd-score between PDAC and healthy controls obtained from the integrated model was statistically significant (*P* value = 8.45E−25) (Fig. 4d), as well as the wd-score difference between PDAC patients with jaundice and those without jaundice (*P* value = 6.28E−03) (Fig. 4e). However, the wd-score of the integrated model failed to classify the resectable and unresectable PDAC samples (Fig. 4f).

Patients with tumor size less than 3 cm (*P* value = 1.9E−02) had significantly higher wd-scores (Table 2). Notably, compared with non-nerve invasive resectable PDAC patients (*n* = 29), patients with nerve invasion had significant higher wd-scores (*P* value = 2.7E−2). No significant differences in vascular invasion or positive lymph node metastasis were found in resectable PDAC patients.

**Table 2:** Comparison of wd-scores (the integrated model) of risk factors and prognosis associated with pancreatic cancer (*n* = 61)

Variable	Variable level	Wd-score	*P* value
Age	≥ 60	− 3.894 ± 0.257	0.383
	< 60	− 4.2126 ± 0254	
Gender	Male	− 3.895 ± 0.230	0.355
	Female	− 4.235 ± 0.289	
Smoking history	Yes	− 4.079 ± 0.375	0.911
	No	− 4.032 ± 0.206	
Alcohol history	Yes	− 4.100 ± 0.314	0.831
	No	− 4.017 ± 0.224	
Chronic disease			
Hypertension	Yes	− 4.094 ± 0.372	0.911
	No	− 4.038 ± 0.201	
Type II diabetes	Yes	− 3.622 ± 0.235	0.118
	No	− 4.139 ± 0.213	
CA199, U/mL	> 37	− 3.915 ± 0.200	0.336
	< 37	− 4.529 ± 0.401	
Jaundice	Yes	− 3.000 ± 0.451	0.006
	No	− 4.357 ± 0.170	
Tumor size	< 3 cm	− 3.751 ± 0.198	0.019
	> 3 cm	− 4.875 ± 0.337	
Primary cancer site	Head	− 4.204 ± 0.252	0.439
	Tail	− 3.920 ± 0.256	
Tumor differentiation	High	/	0.721
	Moderate	− 4.013 ± 0.337	
	Low	− 4.056 ± 0.215	
Metastasis	Yes	− 3.929 ± 0.3508	0.9148
	No	− 4.087 ± 0.2127	

*wd-score* weighted diagnosis score, *CA199* carbohydrate antigen199

*Note*: *P* values were calculated using *t* test

In summary, the integrated prediction model demonstrated higher prediction sensitivity (~ 10% higher), notably in stage I samples (with a sensitivity of 87.5% in the integrated model, and 75.0% and 62.5% in the 5mC and 5hmC model, respectively) (Supplementary Table 5), supporting the prospect of applying the combined cell-free 5mC and 5hmC biomarkers for a more accurate cancer diagnosis.

## Discussion

This study investigated the potential application of epigenetic markers in detecting PDAC, revealing that the 5mC model, 5hmC model, and 5mC-5hmC combined model all showed high prediction accuracy.

Global DNA hypomethylation and a reduction in 5hmC levels are frequently observed in cancer [29, 30]. Our study revealed the global depletion of 5hmC modifications in PDAC cfDNA samples, with the number of 5hmC modification sites decreasing as well. This finding was consistent with the results reported in other cancers, such as colorectal cancer, gastric cancer, and lung cancer [20, 23]. However, global hypermethylation (Supplementary Figure 2C) instead of 5mC depletion was observed in the PDAC cfDNA samples. No significant difference in 5mC peak numbers between the PDAC samples and controls was observed, though the PDAC samples showed a higher median number. Similar results were reported in PDAC tissue [31], for example, the total number of 5mC peaks identified in pancreatic tumor tissues was larger than that in nontumor tissue samples, and more hypermethylated differentially methylated regions (DMRs) were observed in tumor tissues than in nontumor tissues. Likewise, in our study, the number of DMPs hypermethylated in PDAC was much larger than that in controls.

DNA modifications and histone modifications are two important types of epigenetic mechanisms that might be correlated with each other [30]. It has been reported that DNA modification along with histone modification have vital roles in chromosome architecture [32], and DNA methylation might serve as template for histone modification [27]. Increased 5hmC modification and depleted 5mC modification at the permissive histone modification sites in PDAC patients probably indicated elevated expression levels of genes regulated by the elements located in these regions. Deeper investigations combining RNA expression data may further reveal the interactive regulatory mechanism of various types of epigenetic modifications, comprising DNA modifications and histone modifications.

Previous studies focused on gene bodies and defined the DMRs with sliding windows by breaking the reference genome to screen candidate biomarkers [20, 22, 23]. Such methods might lead to statistically significant differences that are not biologically meaningful if few read counts are mapped to the regions in both the cases and controls. The 201 bp fixed-width peak method would reduce the risks since more attention is paid to smaller regions with higher enrichment. Moreover, smaller regions are more convenient and cost-effective considering their further application in clinical practice.

We conducted an exploratory study combining 5mC and 5hmC profiling, and the results suggest that the integration of the two epigenetic approaches improve the diagnostic power as envisioned, particularly the prediction sensitivity in early-stage PDAC samples. The diagnostic effect of the combined model is better than that of the 5hmC model and 5mC model in our study as well as that of models reported by other groups [22, 33] (AUC 0.997 vs 0.94–0.96 and 0.92). In addition, the prediction accuracy is much higher than that of the diagnostic method combining CA199 and Kras mutation (sensitivity 0.94 vs 0.78; specificity 0.95 vs 0.77) [34]. In our study, the CA199 positive patients (> 37 U/L) were 80.3% (Supplementary Table 5), lower than the diagnostic sensitivity of the prediction models.

In the clinic, digital evaluation criteria would be more preferred, so a wd-score was then computed according to the logistic model coefficients and modification level of the corresponding markers for each individual (Supplementary Table 5). The results demonstrated that the wd-scores of both the 5mC and 5hmC markers were higher in patients with jaundice. The possible reason may be that the methylation levels of the epigenetic markers are affected by jaundice, similar to the increase of serum CA199 levels in PDAC patients with jaundice. Nevertheless, the opposite situation is possible. In clinical practice, obstructive jaundice usually occurs when tumor oppresses the common bile duct if the tumor arises in the head of the pancreas. Three 5hmC markers involved in PI (phosphatidylinositol) metabolism was identified, including *PIP5K1A*, *INPP4B*, and *PNPLA7*. It was reported that the ratio of phosphatidylinositol was higher in the plasma of the children with obstructive jaundice than in normal [35]. Further study on non-cancer patients with jaundice are needed to analyze the problem. We speculated that the 5hmC markers identified in this study might be used for the early diagnosis of PDAC given that a significant difference in wd-scores between stage I patients and stage II to IV patients was observed.

Traditionally, age, tumor location, tumor size, tumor grade, TNM stage, and positive lymph node metastasis have been considered important prognostic factors. Although we have not obtained the survival data of the included participants, we compared the wd-scores (from the integrated model) with the aforementioned predictors in the PDAC group (Table 2). Interestingly, we found that patients with tumor size less than 3 cm had higher wd-scores, suggesting further exploration of the mechanism of early tumor progression through gene epigenetic modification.

Nevertheless, there are limitations in this study. For example, a model for early diagnosis was not able to be constructed due to the limited number of stage I patients (*n* = 8). We are dedicated to collecting large-scale samples for further research. In the future, we will adopt this approach for the diagnosis of malignant and benign disease, especially precancerous lesions such as intraductal papillary mucinous neoplasms (IPMNs). The early diagnosis of PDAC is still the top priority of our subsequent work.

## Conclusions

In summary, we have developed a robust noninvasive approach combining epigenetic biomarkers for detecting PDAC. Our study demonstrated that both 5mC and 5hmC biomarkers in cfDNA are effective in PDAC detection, and the 5mC-5hmC integrated model presents increased diagnostic power. Larger numbers of samples and more subtypes of pancreatic diseases are worthy of further investigation.

## Methods

### Study design

This study recruited 72 PDAC patients and 136 healthy controls to investigate the potential diagnostic value of 5mC and 5hmC biomarkers in cfDNA for pancreatic cancer. PDAC was confirmed histopathologically. Healthy participants, enrolled from the community, had normal liver and renal functions, normal cardio-pulmonary function, no history of cancer, and no viral infections. Participants who met the following conditions were excluded: chemotherapy or radiotherapy for malignant tumors, metastatic PDAC, or PDAC with other cancer.

For 5hmC profiling, 5 samples were excluded because of the poor quality or low quantity of the sample. Five samples did not have 5mC profiling data due to an insufficient amount of cfDNA. All of the 136 healthy control samples were applied to 5hmC sequencing and cfMeDIP-seq, but only 97 of them yielded 5mC data when the analysis started.

### Clinical sample collection and sample processing

Blood was collected into BD Vacutainer® EDTA tubes (Becton, Dickinson and Company, Cat# 367525). Within 2 h, plasma was separated from blood by centrifugation at 1600×*g* at 4 °C for 10 min followed by 16000×*g* at 4 °C for 10 min. Then, cfDNA was extracted with a QIAseq cfDNA Extraction kit (a part of the QIAseq All-in-one Kit, Cat. No. 180025) and quantified by a Qubit fluorometer (Life Technologies). CfDNA (10–30 ng) was applied for library construction: adaptor ligation was facilitated using the QIAseq cfDNA Library Kit (a part of the QIAseq All-in-one Kit, Cat. No. 180025) following the protocol provided by the manufacturer, with spike-in controls added (0.01 pg of each amplicon per 10 ng of cfDNA). The spike-in control, including 3 distinct lambda DNA amplification products (~ 180 bp) (one without modification and the other two with 5mC and 5hmC modifications) were prepared following the method described by Chun-Xiao Song et al [23].

### Methylome profiling

Ligated cfDNA (5–10 ng) was applied for methylome profiling following the previously published cfMeDIP-seq protocol [21, 22] with minor modifications. We used the spike-in controls mentioned above to roughly assess the 5mC enrichment ratio instead of the methylated DNA (meDNA) and unmethylated DNA (unDNA) spike-in controls used in cfMeDIP-seq. In brief, cfDNA libraries with the spike-in control and the filler DNA, as well as Buffer A and Buffer B provided in the Magnetic Methylated DNA Immunoprecipitation Kit (Diagenode, C02010021), were incubated at 95 °C for 10 min and immediately chilled on ice for 10 min. Then, 75 μl of incubation mix was incubated with anti-5mC antibody (Magnetic Methylated DNA Immunoprecipitation Kit, Diagenode, C02010021) on a rotating wheel at 4 °C for 17 h, followed by purification with the Magnetic IPure kit v2 (Diagenode, Cat. No. C03010015). The final libraries were amplified with QIAGEN HiFi PCR Master Mix, 2x and Primer Mix Illumina Libr. Amp (QIAseq All-in-one Kit, Cat. No. 180025) as follows: 98 °C for 2 min, followed by 9–12 cycles of 98 °C for 20 s, 60 °C for 30 s, 72 °C for 30 s, and a final extension at 72 °C for 1 min. Afterward, the amplified libraries were purified using 0.8× Beckman Agencourt AMPure XP beads (Cat. No. A63881) and quantified by a Qubit fluorometer (Life Technologies). Pair-end 150 bp sequencing was performed on the Illumina NovaSeq 6000 system by Novogene Co., Ltd. (Beijing).

### 5hmC sequencing

5hmC profiling was performed using the method reported previously—the cfDNA 5hmC sequencing method based on selective chemical labeling (hMe-Seal) [23]. All procedures followed the protocol described in the paper. In brief, cfDNA ligated with sequencing adaptors was incubated in a 25 μl reaction solution containing HEPES buffer (50 mM, pH 8.0), MgCl_2_ (25 mM), 60 μM N_3_-UDP-Glc (Active Motif, Carlsbad, CA, USA), and 12.5 U β-glucosyltransferase (NEB) for 2 h at 37 °C. Then, 2.5 μl of DBCO-PEG_4_-biotin (Sigma) was directly added and incubated for 2 h at 37 °C. Next, 10 μg of sheared salmon sperm DNA (Life Technologies) was added. Subsequently, a Micro Bio-Spin 30 Column (Bio-Rad) was used to purify the DNA following the instructions, and the volume was adjusted to 25 μl. Afterward, purified DNA was incubated with 5 μl of C1 streptavidin beads (Life Technologies, USA) in buffer 1 (5 mM Tris pH 7.5, 0.5 mM EDTA, 1 M NaCl, and 0.2% Tween 20) for 30 min. The beads were subsequently subjected to three 5-min washes each with buffer 1, buffer 2 (buffer 1 without NaCl), buffer 3 (buffer 1 with pH 9), and buffer 4 (buffer 3 without NaCl). The beads were then resuspended in water and amplified with 9–12 cycles of PCR amplification (initial denaturing at 98 °C for 45 s, followed by 9–12 cycles of denaturing at 98 °C for 15 s, annealing at 60 °C for 30 s, extension at 72 °C for 30 s, and a final extension at 72 °C for 5 min). The amplified product was purified using AMPure XP beads. Pair-end 150 bp sequencing was performed on the Illumina NovaSeq 6000 platform by Novogene Co., Ltd. (Beijing).

### Data processing

For 5hmC and 5mC sequencing data alignment, the following methods were applied. The data quality of raw FASTQ files was checked by FastQC (version 0.11.8). The adapters of the raw FASTQ files were removed by Trimmomatic (version 0.38). Processed sequencing reads were aligned to hg19 and spike-in DNA using Bowtie2 (version 2.3.4.3) [36] with default parameters. The generated SAM files were filtered by SAMtools (version 1.9) [37] with the parameter settings of “-f 2 -F 1548 -q 30” to include high-quality, properly paired reads and then were converted to BAM format. Picard (version 2.18.23) (http://broadinstitute.github.io/picard) was employed to sort and index the filtered SAM files and to ensure the removal of duplicate reads before subsequent analysis. There were three types of spike-in DNA sequences, and capture efficiency, as a quality control measurement for 5hmC and 5mC, was calculated as the counts of reads aligned to a type-specific spike-in DNA divided by the counts of reads aligned to the total spike-in DNA.

### Peak detection

5hmC and 5mC sequencing data peak detection was accomplished with the following steps. MACS2 (version 2.1.2) [38] was utilized to call peaks for each sequencing dataset. To obtain a high-confidence consensus peak list while considering differences in the sequencing depth and sample status (PDAC or healthy), a peak calling procedure was adopted as previously described [39]. In brief, the raw peak list within a single sample generated by MACS2 was processed by first extending 100 bp on either side of the peak summits, then normalizing MACS2 peak scores as “score per million” and finally removing overlapping peaks with an iterative removal procedure. Next, peaks defined as reproducible peaks with score per million > = 5 were merged into group-specific (PDAC-specific or healthy-specific) peak lists by the same iterative procedure, and at last, the final consensus peak list was generated. Reproducible peaks are peaks emerging in at least N% of cancer samples or healthy controls. Following the procedure mentioned above, nine consensus peak lists were generated with a set of values for N (N range, 10 ~ 90 with 10 increments). For each consensus peak list, we could obtain a zero-one matrix with rows representing consensus peaks and columns representing all samples, where one means a sample has a peak overlapping with a consensus peak and zero means no peak. Then, Fisher’s exact test was applied to determine peaks with the most striking differences between PDAC samples and healthy controls. For 5mC data, *N* = 10 resulted in the most significant peaks, and it was 30 in 5hmC data.

In particular, this peak list merging process was applied for 5hmC and 5mC data separately. All the peaks involved in the ENCODE hg19 blacklist [40], peaks that extend beyond any ends of chromosomes and peaks on chromosomes X, Y or on the mitochondrial genome, were filtered.

Normalized pileup tracks generated by MACS2 were converted to bigwig format using bedGraphToBigWig from the UCSC Genome Browser and then put into the IGV for visualization [25, 26].

Bedtools (version 2.25.0) [41] was used to obtain the fragment counts of the final consensus peak list in each sample, and 5hmC or 5mC FPKM was then calculated. Differential peak analysis of the PDAC samples and healthy controls in the 5hmC and 5mC data was performed using Student’s *t* test (function *t* test in R), as well as differential analysis of PDAC samples between 5mC and 5hmC data. For the *t-*SNE plot, the FPKM of the consensus peak list was used as input, and the seed was set to 40 (perplexity was 52).

### Peak annotation

To compare the distribution of peak numbers in PDAC and healthy samples for both 5mC and 5hmC data, the Wilcoxon test was used; the same was done for the comparison between 5mC and 5hmC data in PDAC samples. The genomic element distribution of peaks was determined by the percentage of peaks overlapping each element through BEDtools (> 1 bp). The enrichment analysis of peak occupancy in genomic features was assessed by the odds ratio. Ngs.plot [42] was used to characterize metagene profiles. Gene Ontology (GO) analysis was performed by the ReactomePA R package (version 1.28.0) [43].

ChIP-Seq files of H3K4me1, H3K4me3, H3K36me3, H3K27ac, and H3K27me3 of the pancreatic cancer cell line PANC-1 provided by ENCODE were downloaded with the following identifiers: ENCFF520QXI, ENCFF213GUQ, ENCFF922RLL, ENCFF629BRY, and ENCFF915XVA [44]. BEDtools was adopted to obtain the percentage of peaks overlapping each histone modification region (> 1 bp).

### Biomarker selection and model construction

The elastic-net regularization on a logistic linear regression model in the glmnet R package (version 2.0-18) [45] was chosen to establish prediction models. To filter more effective biomarkers for distinguishing PDAC samples from healthy controls, the following procedure was applied.

For the 5mC data set, we randomly split 70% of the healthy samples (*n* = 67) and 75% of the PDAC samples (*n* = 50) into the training set and the remaining samples into the validation set. To avoid overfitting, 5 rounds of 10-fold cross-validation was performed (Figure S8). The details were as follows: the training set was randomly divided into five folds, four of which were selected as the training subset, and the remaining one was the test subset. In each training subset, the DhMPs between PDAC samples and healthy controls with false discovery rate (FDR) < 0.01 and |log2FC| > 0.8 (*t* test) remained as candidates. Then, we performed 100 repeats to further select markers using the elastic-net model, and a panel of DhMPs in each training subset that appeared in at least 95% of the iterations was retained. Thus, 10-fold cross-validation was repeated 100 times each round. Finally, the final markers observed in at least 3 rounds were used to build the final prediction model in the training set, and this model was utilized to predict the validation samples. The *α* was selected with maximize accuracy in the validation set over a grid of values from 0.1 to 0.9.

For the 5hmC data set, 75% of the PDAC samples (*n* = 39) and 75% of the healthy samples (*n* = 79) were randomly divided into the training set, and the remaining samples were placed into the validation set. The same procedure as that in the 5mC modeling pipeline was adopted, except for *α*, which was 0.1, and the criteria for the *t* test, which was altered to *P* value < 0.001 and |log2FC| > 0.8.

For the 5mC and 5hmC conjoint analysis, samples with both 5mC and 5hmC data were included. The training set consisted of 75% of the healthy samples (*n* = 64) and 75% of the PDAC samples (*n* = 45), and the validation set consisted of the remaining data. The twenty-four 5mC biomarkers and the twenty-seven 5hmC biomarkers identified previously were combined for elastic-net model training (Figure S10).

The wd-score was calculated for each sample according to the biomarker model coefficients as follows:

$$ Wd- score= sum\ \left( coef\ (k)\ast FPKM\ (k)\right),\mathrm{where}\ k\ \mathrm{represents}\ \mathrm{the}\ \mathrm{marker} $$

### Statistical analysis

Descriptive statistics of the characteristics of the participants were calculated using SPSS (v23.0, IBM, Armonk, NY, USA) as well as the chi-squared test for categorical variables. All tests were two-sided, and *P* values < 0.05 were considered statistically significant.

We also assessed the associations of the wd-score with prognostic indicators such as age over 60, male sex, smoking status, alcohol consumption, CA199 over 37 U/ml, tumor size over 3 cm, tumor location (head, body, and tail), tumor differentiation (high, moderate and low), and the occurrence of metastasis in PDAC patients using *t* test to evaluate the application value of the wd-score for the prognosis prediction of PDAC.

Statistical analyses were performed in R 3.6.3. The Wilcoxon-Mann-Whitney test was used to compare different groups except that ANOVA was applied to compare the wd-scores of 5mC, 5hmC, and the integrated 5mC-5hmC model in different AJCC-staged pancreatic cancer patients. Raw *P* values were corrected by Benjamini and Hochberg correction. The R packages R*t*SNE (version 0.15) [46] and pheatmap (version 1.0.12) were used for dimension reduction and clustering analysis. The glmnet package was utilized to construct prediction models. The roc function of the R package pROC (version 1.15.3) was used to generate receiver operating characteristic (ROC) curves and calculate the AUC.

## Supplementary information

**Additional file 1: Supplementary Figure 1.** Percentage of reads mapped to the spike-in DNA. A. The 5mC spike-in DNA is specifically enriched in the 5mC libraries. B. The 5hmC spike-in DNA is specifically enriched in the 5hmC libraries. Error bars indicate Standard Deviation (SD). **Supplementary Figure 2.** Global change in 5mC and 5hmC level in PDAC. A. Boxplot of 5mC peak numbers from healthy controls and PDAC samples shows no significant difference. B. Boxplot of 5hmC peak numbers from healthy controls and PDAC samples shows significant larger numbers of peaks in PDAC samples. C. Genome browser view of the cell-free 5mC distribution in a 5 mb region in chromosome 8. D. Genome browser view of the cell-free 5hmC distribution in a 3 mb region in chromosome 7. The overlapping tracks of healthy and PDAC are shown in line plot. *P < 0.05, **P < 0.01, ***P < 0.001, ****P < 1e−5, Wilcoxon test. PDAC, pancreatic ductal adenocarcinoma. **Supplementary Figure 3.** Genomic distribution of 5mC and 5hmC peaks. A. 5mC distribution in genomic features. B. Enrichment of 5mC peaks overlapping with distinct genomic elements. C. 5hmC peak distribution in genomic features. D. Enrichment of 5hmC peaks overlapping with distinct genomic elements. PDAC, pancreatic ductal adenocarcinoma; CDS, Coding DNA Sequence; 3′UTR, 3′untranslated region; 5′UTR, 5′untranslated region. **Supplementary Figure 4.** Comparison of the 5mC and 5hmC peaks. A. Venn diagram of overlap between 5mC and 5hmC peaks. B. Venn diagram of overlap between genes with 5mC modifications and genes with 5hmC modifications. **Supplementary Figure 5.** GO term enrichment analysis of specifically modified genes. A. 5mC-specific genes. B. 5hmC-specific genes. PDAC, pancreatic ductal adenocarcinoma. **Supplementary Figure 6.** Genome browser views of examples of specifically modified genes. A. *ME1* gene in chromosome 6: 84,095–84,140 kb. B. *PACRG* gene in chromosome 6: 163,716–163,734 kb. C. *FYN* gene in chromosome 6: 112,132–112,148 kb. D. *RALB* gene in chromosome 2: 121,000–121,030 kb. *ME*1, malic enzyme 1; *PACRG*, parkin coregulated; *FYN*, FYN proto-oncogene; *RALB*, RAS like proto-oncogene B. **Supplementary Figure 7.** T-SNE analysis of 5mC FPKM. A. T-SNE plot of 5mC FPKM from PDAC and healthy samples in distinct batches after removing batch effect. B. T-SNE plot of 5mC FPKM from PDAC samples and healthy samples. PDAC, pancreatic ductal adenocarcinoma. **Supplementary Figure 8.** Flow chart of 5mC model construction. **Supplementary Figure 9.** T-SNE analysis of 5hmC FPKM. A. T-SNE plot of 5hmC FPKM from PDAC and healthy samples in distinct batches. B. T-SNE plot of 5hmC FPKM from PDAC and healthy samples. PDAC, pancreatic ductal adenocarcinoma. **Supplementary Figure 10.** Flow chart of 5hmC model construction. **Supplementary Figure 11.** Performance of the 5hmC model in distinguishing the subgroups of PDAC patients. A. Boxplot of the wd-scores in the resectable PDAC patients and unresectable PDAC patients. B. Boxplot of the wd-scores in PDAC patients with jaundice and those without jaundice. *P < 0.05, **P < 0.01, ***P < 0.001, ****P < 1e−5, Wilcoxon test.

**Additional file 2: Supplementary Table 1.** Mapping summary of cfDNA 5mC sequencing data. **Supplementary Table 2.** Mapping summary of cfDNA 5hmC sequencing results. **Supplementary Table 3.** Differentially methylated peaks identified by *t* test. **Supplementary Table 4.** List of 5mC markers used in model construction. **Supplementary Table 5.** Wd-scores of PDAC patients derived from distinct models. **Supplementary Table 6.** Differentially hydroxymethylated peaks identified by *t* test. **Supplementary Table 7.** List of 5hmC markers used in model construction.

## Data Availability

https://pms.cd120.com/PDAC/index.html

## Abbreviations

5hmC: 5-Hydroxymethylcytosine
5mC: 5-Methylcytosine
AJCC: American Joint Committee on Cancer
AUC: Area under the curve
cfDNA: Cell-free DNA
cfMeDIP-seq: Cell-free methylated DNA immunoprecipitation and high-throughput sequencing
ctDNA: Circulating tumor DNA
DhMPs: Differentially hydroxymethylated peaks
DMPs: Differentially methylated peaks
FPKM: Fragments per kilobase of gene per million mapped reads
hMe-Seal: 5hmC-selective chemical labeling
IGV: Integrative Genomics Viewer
PDAC: Pancreatic ductal adenocarcinoma
*t-*SNE: t-distributed stochastic neighbor embedding analysis
wd-score: Weighted diagnosis score
