# Supplementary figures and images for: Integrated epigenetic biomarkers in circulating cell-free DNA as a robust classifier for pancreatic cancer
Source: Clin Epigenetics. 2020 Jul 23;12:112. doi: 10.1186/s13148-020-00898-2 (PMC7376965; doi:10.1186/s13148-020-00898-2)

**A**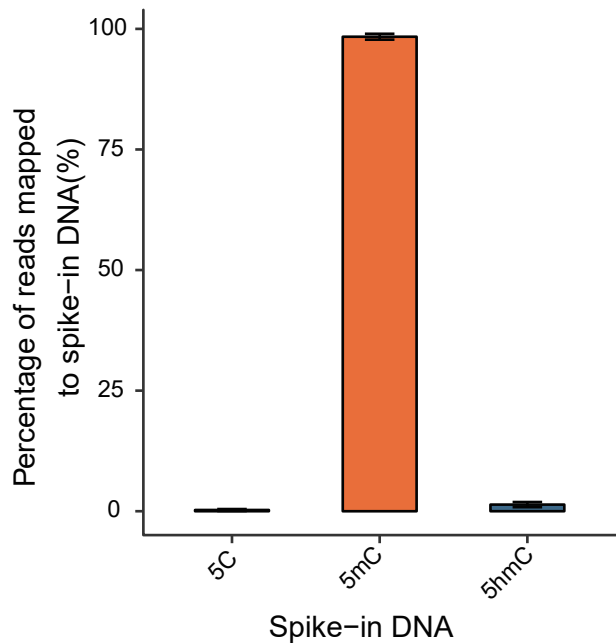**B**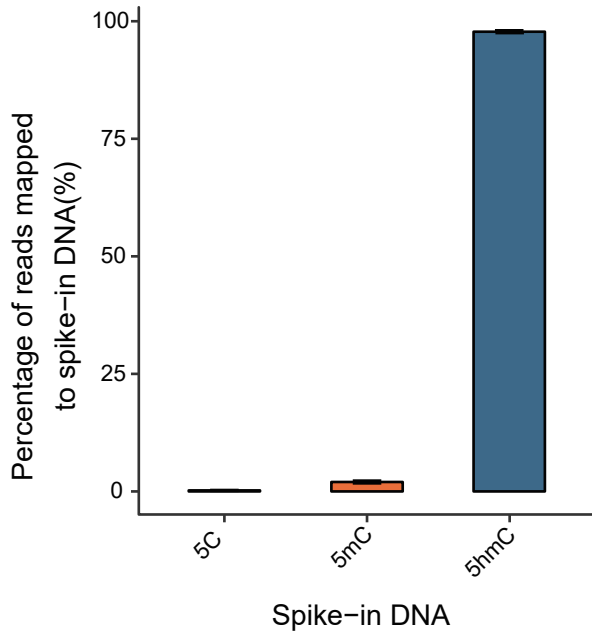

Supplement: Supplementary file 1 — Additional file 1: Supplementary Figure 1. Percentage of reads mapped to the spike-in DNA. A. The 5mC spike-in DNA is specifically enriched in the 5mC libraries. B. The 5hmC spike-in DNA is specifically enriched in the 5hmC libraries. Error bars indicate Standard Deviation (SD). Supplementary Figure 2. Global change in 5mC and 5hmC level in PDAC. A. Boxplot of 5mC peak numbers from healthy controls and PDAC samples shows no significant difference. B. Boxplot of 5hmC peak numbers from healthy controls and PDAC samples shows significant larger numbers of peaks in PDAC samples. C. Genome browser view of the cell-free 5mC distribution in a 5 mb region in chromosome 8. D. Genome browser view of the cell-free 5hmC distribution in a 3 mb region in chromosome 7. The overlapping tracks of healthy and PDAC are shown in line plot. *P < 0.05, **P < 0.01, ***P < 0.001, ****P < 1e−5, Wilcoxon test. PDAC, pancreatic ductal adenocarcinoma. Supplementary Figure 3. Genomic distribution of 5mC and 5hmC peaks. A. 5mC distribution in genomic features. B. Enrichment of 5mC peaks overlapping with distinct genomic elements. C. 5hmC peak distribution in genomic features. D. Enrichment of 5hmC peaks overlapping with distinct genomic elements. PDAC, pancreatic ductal adenocarcinoma; CDS, Coding DNA Sequence; 3′UTR, 3′untranslated region; 5′UTR, 5′untranslated region. Supplementary Figure 4. Comparison of the 5mC and 5hmC peaks. A. Venn diagram of overlap between 5mC and 5hmC peaks. B. Venn diagram of overlap between genes with 5mC modifications and genes with 5hmC modifications. Supplementary Figure 5. GO term enrichment analysis of specifically modified genes. A. 5mC-specific genes. B. 5hmC-specific genes. PDAC, pancreatic ductal adenocarcinoma. Supplementary Figure 6. Genome browser views of examples of specifically modified genes. A. ME1 gene in chromosome 6: 84,095–84,140 kb. B. PACRG gene in chromosome 6: 163,716–163,734 kb. C. FYN gene in chromosome 6: 112,132–112,148 kb. D. RALB gene i [file 13148_2020_898_MOESM1_ESM.zip › Figure S1.pdf]

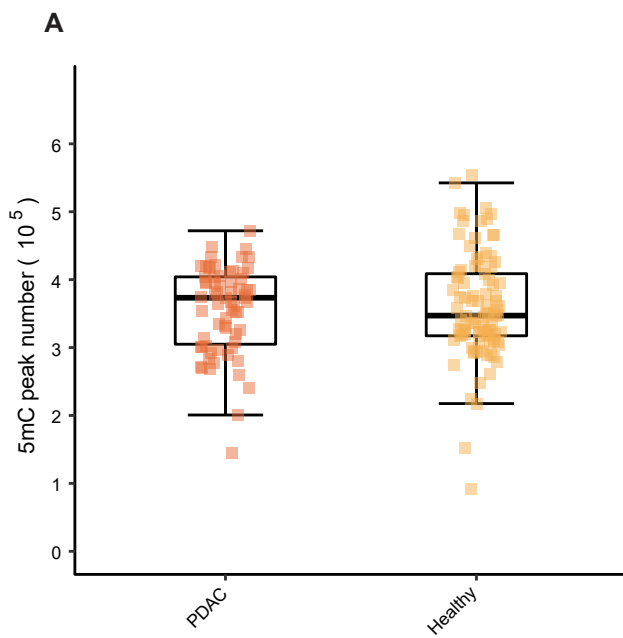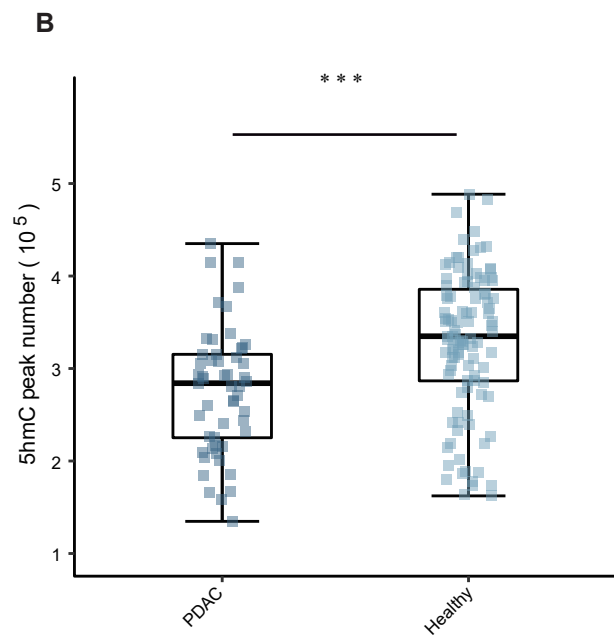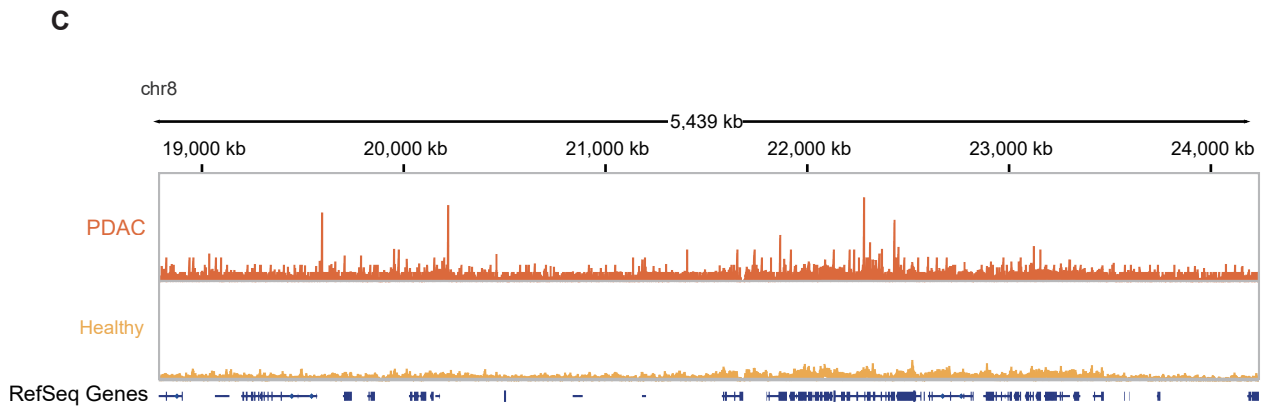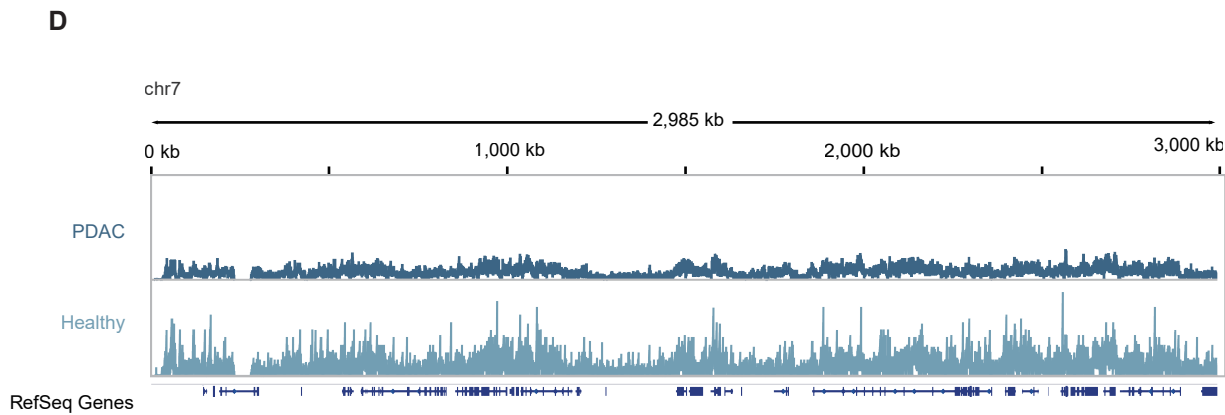

Supplement: Supplementary file 1 — Additional file 1: Supplementary Figure 1. Percentage of reads mapped to the spike-in DNA. A. The 5mC spike-in DNA is specifically enriched in the 5mC libraries. B. The 5hmC spike-in DNA is specifically enriched in the 5hmC libraries. Error bars indicate Standard Deviation (SD). Supplementary Figure 2. Global change in 5mC and 5hmC level in PDAC. A. Boxplot of 5mC peak numbers from healthy controls and PDAC samples shows no significant difference. B. Boxplot of 5hmC peak numbers from healthy controls and PDAC samples shows significant larger numbers of peaks in PDAC samples. C. Genome browser view of the cell-free 5mC distribution in a 5 mb region in chromosome 8. D. Genome browser view of the cell-free 5hmC distribution in a 3 mb region in chromosome 7. The overlapping tracks of healthy and PDAC are shown in line plot. *P < 0.05, **P < 0.01, ***P < 0.001, ****P < 1e−5, Wilcoxon test. PDAC, pancreatic ductal adenocarcinoma. Supplementary Figure 3. Genomic distribution of 5mC and 5hmC peaks. A. 5mC distribution in genomic features. B. Enrichment of 5mC peaks overlapping with distinct genomic elements. C. 5hmC peak distribution in genomic features. D. Enrichment of 5hmC peaks overlapping with distinct genomic elements. PDAC, pancreatic ductal adenocarcinoma; CDS, Coding DNA Sequence; 3′UTR, 3′untranslated region; 5′UTR, 5′untranslated region. Supplementary Figure 4. Comparison of the 5mC and 5hmC peaks. A. Venn diagram of overlap between 5mC and 5hmC peaks. B. Venn diagram of overlap between genes with 5mC modifications and genes with 5hmC modifications. Supplementary Figure 5. GO term enrichment analysis of specifically modified genes. A. 5mC-specific genes. B. 5hmC-specific genes. PDAC, pancreatic ductal adenocarcinoma. Supplementary Figure 6. Genome browser views of examples of specifically modified genes. A. ME1 gene in chromosome 6: 84,095–84,140 kb. B. PACRG gene in chromosome 6: 163,716–163,734 kb. C. FYN gene in chromosome 6: 112,132–112,148 kb. D. RALB gene i [file 13148_2020_898_MOESM1_ESM.zip › Figure S2.pdf]

**A**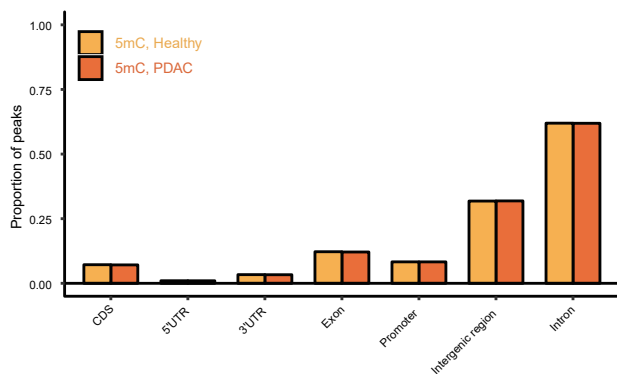**B**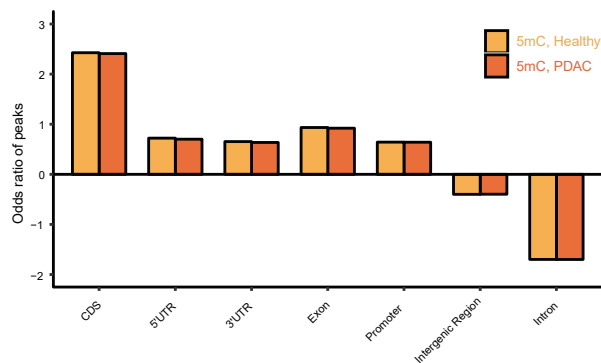**C**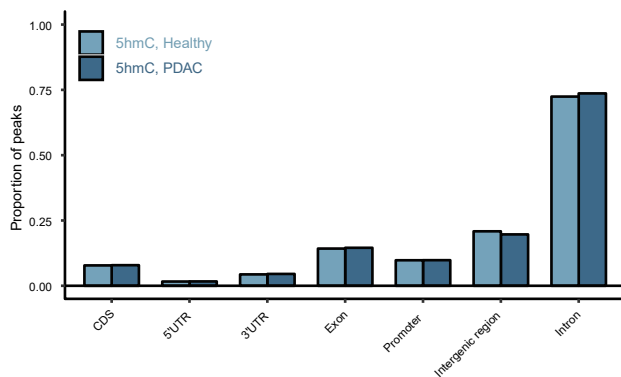**D**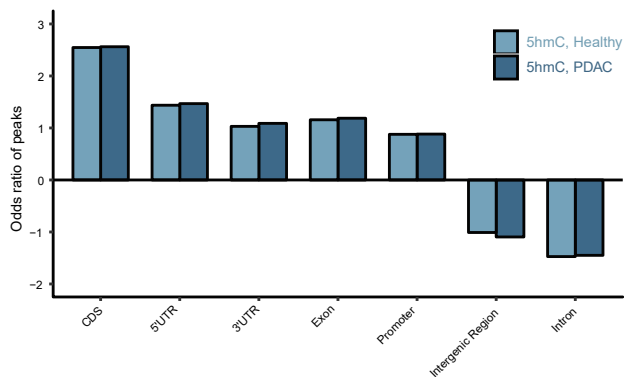

Supplement: Supplementary file 1 — Additional file 1: Supplementary Figure 1. Percentage of reads mapped to the spike-in DNA. A. The 5mC spike-in DNA is specifically enriched in the 5mC libraries. B. The 5hmC spike-in DNA is specifically enriched in the 5hmC libraries. Error bars indicate Standard Deviation (SD). Supplementary Figure 2. Global change in 5mC and 5hmC level in PDAC. A. Boxplot of 5mC peak numbers from healthy controls and PDAC samples shows no significant difference. B. Boxplot of 5hmC peak numbers from healthy controls and PDAC samples shows significant larger numbers of peaks in PDAC samples. C. Genome browser view of the cell-free 5mC distribution in a 5 mb region in chromosome 8. D. Genome browser view of the cell-free 5hmC distribution in a 3 mb region in chromosome 7. The overlapping tracks of healthy and PDAC are shown in line plot. *P < 0.05, **P < 0.01, ***P < 0.001, ****P < 1e−5, Wilcoxon test. PDAC, pancreatic ductal adenocarcinoma. Supplementary Figure 3. Genomic distribution of 5mC and 5hmC peaks. A. 5mC distribution in genomic features. B. Enrichment of 5mC peaks overlapping with distinct genomic elements. C. 5hmC peak distribution in genomic features. D. Enrichment of 5hmC peaks overlapping with distinct genomic elements. PDAC, pancreatic ductal adenocarcinoma; CDS, Coding DNA Sequence; 3′UTR, 3′untranslated region; 5′UTR, 5′untranslated region. Supplementary Figure 4. Comparison of the 5mC and 5hmC peaks. A. Venn diagram of overlap between 5mC and 5hmC peaks. B. Venn diagram of overlap between genes with 5mC modifications and genes with 5hmC modifications. Supplementary Figure 5. GO term enrichment analysis of specifically modified genes. A. 5mC-specific genes. B. 5hmC-specific genes. PDAC, pancreatic ductal adenocarcinoma. Supplementary Figure 6. Genome browser views of examples of specifically modified genes. A. ME1 gene in chromosome 6: 84,095–84,140 kb. B. PACRG gene in chromosome 6: 163,716–163,734 kb. C. FYN gene in chromosome 6: 112,132–112,148 kb. D. RALB gene i [file 13148_2020_898_MOESM1_ESM.zip › Figure S3.pdf]

**A**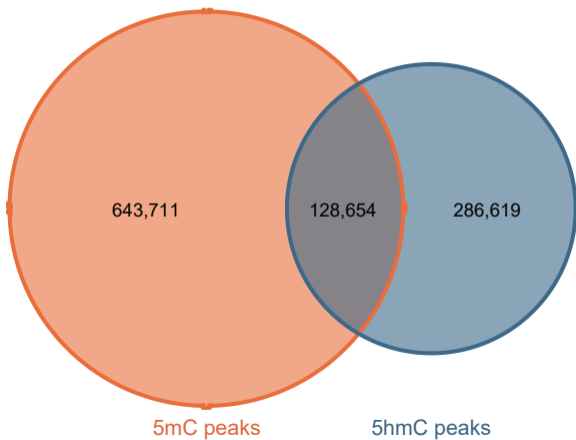**B**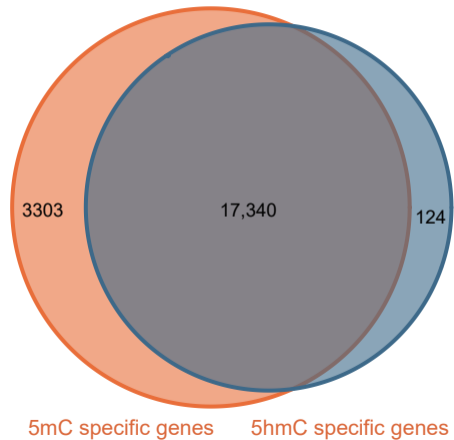

Supplement: Supplementary file 1 — Additional file 1: Supplementary Figure 1. Percentage of reads mapped to the spike-in DNA. A. The 5mC spike-in DNA is specifically enriched in the 5mC libraries. B. The 5hmC spike-in DNA is specifically enriched in the 5hmC libraries. Error bars indicate Standard Deviation (SD). Supplementary Figure 2. Global change in 5mC and 5hmC level in PDAC. A. Boxplot of 5mC peak numbers from healthy controls and PDAC samples shows no significant difference. B. Boxplot of 5hmC peak numbers from healthy controls and PDAC samples shows significant larger numbers of peaks in PDAC samples. C. Genome browser view of the cell-free 5mC distribution in a 5 mb region in chromosome 8. D. Genome browser view of the cell-free 5hmC distribution in a 3 mb region in chromosome 7. The overlapping tracks of healthy and PDAC are shown in line plot. *P < 0.05, **P < 0.01, ***P < 0.001, ****P < 1e−5, Wilcoxon test. PDAC, pancreatic ductal adenocarcinoma. Supplementary Figure 3. Genomic distribution of 5mC and 5hmC peaks. A. 5mC distribution in genomic features. B. Enrichment of 5mC peaks overlapping with distinct genomic elements. C. 5hmC peak distribution in genomic features. D. Enrichment of 5hmC peaks overlapping with distinct genomic elements. PDAC, pancreatic ductal adenocarcinoma; CDS, Coding DNA Sequence; 3′UTR, 3′untranslated region; 5′UTR, 5′untranslated region. Supplementary Figure 4. Comparison of the 5mC and 5hmC peaks. A. Venn diagram of overlap between 5mC and 5hmC peaks. B. Venn diagram of overlap between genes with 5mC modifications and genes with 5hmC modifications. Supplementary Figure 5. GO term enrichment analysis of specifically modified genes. A. 5mC-specific genes. B. 5hmC-specific genes. PDAC, pancreatic ductal adenocarcinoma. Supplementary Figure 6. Genome browser views of examples of specifically modified genes. A. ME1 gene in chromosome 6: 84,095–84,140 kb. B. PACRG gene in chromosome 6: 163,716–163,734 kb. C. FYN gene in chromosome 6: 112,132–112,148 kb. D. RALB gene i [file 13148_2020_898_MOESM1_ESM.zip › Figure S4.pdf]

**A**

## PDAC 5mC specific gene enrich pathway

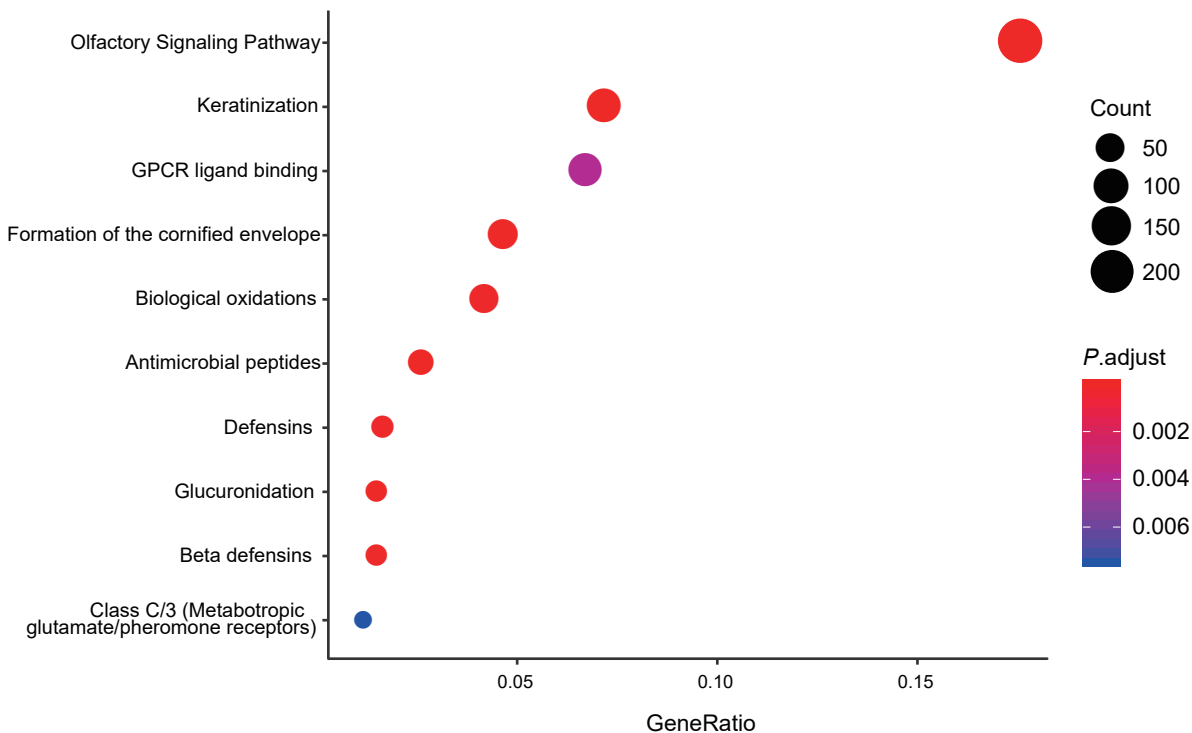**B**

## PDAC 5hmC specific gene enrich pathway

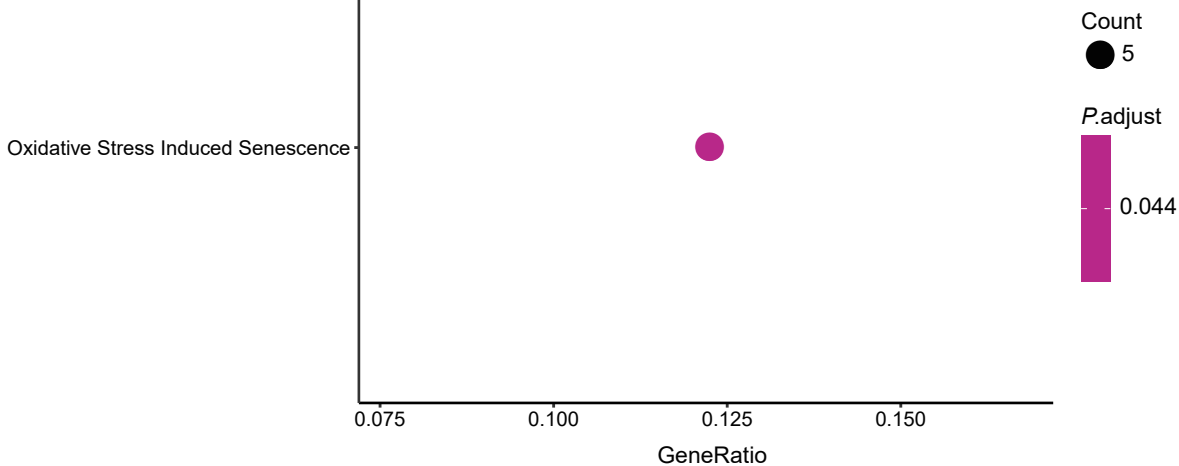

Supplement: Supplementary file 1 — Additional file 1: Supplementary Figure 1. Percentage of reads mapped to the spike-in DNA. A. The 5mC spike-in DNA is specifically enriched in the 5mC libraries. B. The 5hmC spike-in DNA is specifically enriched in the 5hmC libraries. Error bars indicate Standard Deviation (SD). Supplementary Figure 2. Global change in 5mC and 5hmC level in PDAC. A. Boxplot of 5mC peak numbers from healthy controls and PDAC samples shows no significant difference. B. Boxplot of 5hmC peak numbers from healthy controls and PDAC samples shows significant larger numbers of peaks in PDAC samples. C. Genome browser view of the cell-free 5mC distribution in a 5 mb region in chromosome 8. D. Genome browser view of the cell-free 5hmC distribution in a 3 mb region in chromosome 7. The overlapping tracks of healthy and PDAC are shown in line plot. *P < 0.05, **P < 0.01, ***P < 0.001, ****P < 1e−5, Wilcoxon test. PDAC, pancreatic ductal adenocarcinoma. Supplementary Figure 3. Genomic distribution of 5mC and 5hmC peaks. A. 5mC distribution in genomic features. B. Enrichment of 5mC peaks overlapping with distinct genomic elements. C. 5hmC peak distribution in genomic features. D. Enrichment of 5hmC peaks overlapping with distinct genomic elements. PDAC, pancreatic ductal adenocarcinoma; CDS, Coding DNA Sequence; 3′UTR, 3′untranslated region; 5′UTR, 5′untranslated region. Supplementary Figure 4. Comparison of the 5mC and 5hmC peaks. A. Venn diagram of overlap between 5mC and 5hmC peaks. B. Venn diagram of overlap between genes with 5mC modifications and genes with 5hmC modifications. Supplementary Figure 5. GO term enrichment analysis of specifically modified genes. A. 5mC-specific genes. B. 5hmC-specific genes. PDAC, pancreatic ductal adenocarcinoma. Supplementary Figure 6. Genome browser views of examples of specifically modified genes. A. ME1 gene in chromosome 6: 84,095–84,140 kb. B. PACRG gene in chromosome 6: 163,716–163,734 kb. C. FYN gene in chromosome 6: 112,132–112,148 kb. D. RALB gene i [file 13148_2020_898_MOESM1_ESM.zip › Figure S5.pdf]

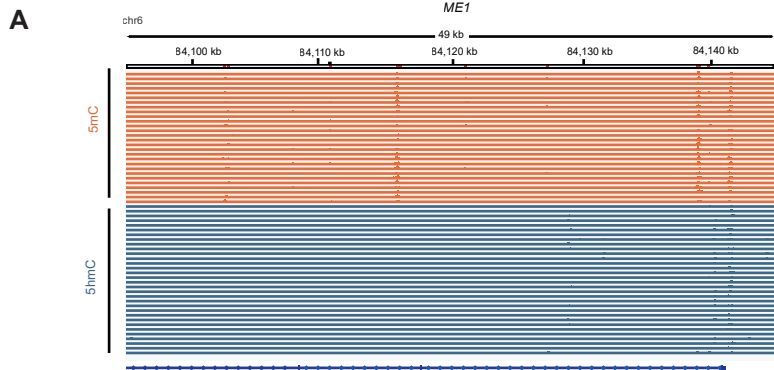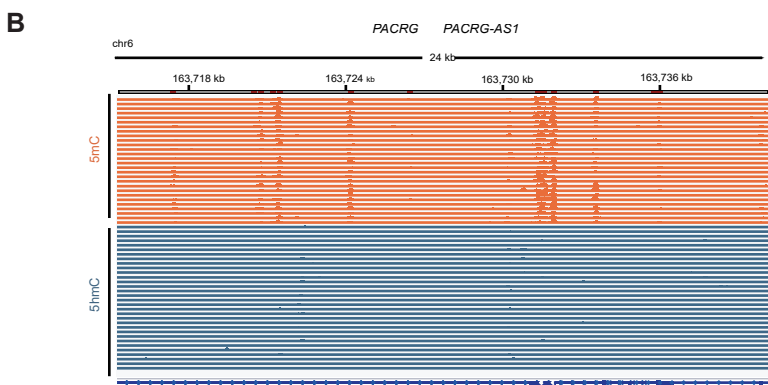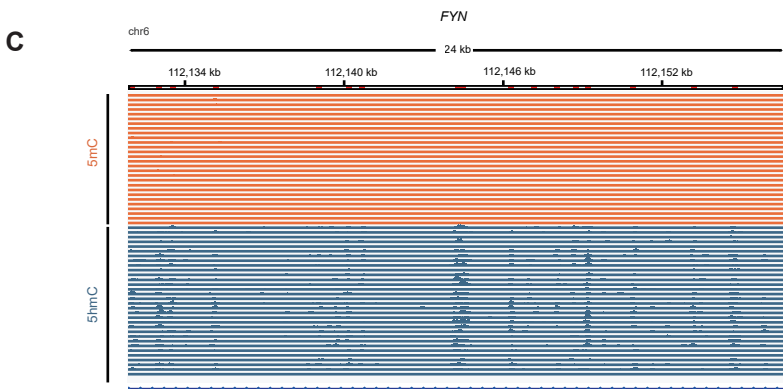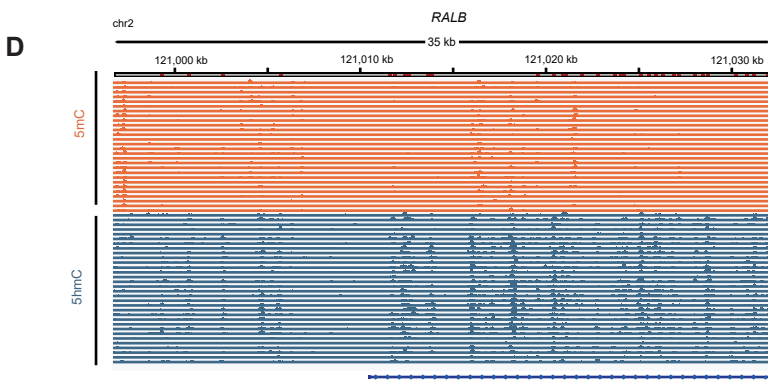

Supplement: Supplementary file 1 — Additional file 1: Supplementary Figure 1. Percentage of reads mapped to the spike-in DNA. A. The 5mC spike-in DNA is specifically enriched in the 5mC libraries. B. The 5hmC spike-in DNA is specifically enriched in the 5hmC libraries. Error bars indicate Standard Deviation (SD). Supplementary Figure 2. Global change in 5mC and 5hmC level in PDAC. A. Boxplot of 5mC peak numbers from healthy controls and PDAC samples shows no significant difference. B. Boxplot of 5hmC peak numbers from healthy controls and PDAC samples shows significant larger numbers of peaks in PDAC samples. C. Genome browser view of the cell-free 5mC distribution in a 5 mb region in chromosome 8. D. Genome browser view of the cell-free 5hmC distribution in a 3 mb region in chromosome 7. The overlapping tracks of healthy and PDAC are shown in line plot. *P < 0.05, **P < 0.01, ***P < 0.001, ****P < 1e−5, Wilcoxon test. PDAC, pancreatic ductal adenocarcinoma. Supplementary Figure 3. Genomic distribution of 5mC and 5hmC peaks. A. 5mC distribution in genomic features. B. Enrichment of 5mC peaks overlapping with distinct genomic elements. C. 5hmC peak distribution in genomic features. D. Enrichment of 5hmC peaks overlapping with distinct genomic elements. PDAC, pancreatic ductal adenocarcinoma; CDS, Coding DNA Sequence; 3′UTR, 3′untranslated region; 5′UTR, 5′untranslated region. Supplementary Figure 4. Comparison of the 5mC and 5hmC peaks. A. Venn diagram of overlap between 5mC and 5hmC peaks. B. Venn diagram of overlap between genes with 5mC modifications and genes with 5hmC modifications. Supplementary Figure 5. GO term enrichment analysis of specifically modified genes. A. 5mC-specific genes. B. 5hmC-specific genes. PDAC, pancreatic ductal adenocarcinoma. Supplementary Figure 6. Genome browser views of examples of specifically modified genes. A. ME1 gene in chromosome 6: 84,095–84,140 kb. B. PACRG gene in chromosome 6: 163,716–163,734 kb. C. FYN gene in chromosome 6: 112,132–112,148 kb. D. RALB gene i [file 13148_2020_898_MOESM1_ESM.zip › Figure S6.pdf]

**A**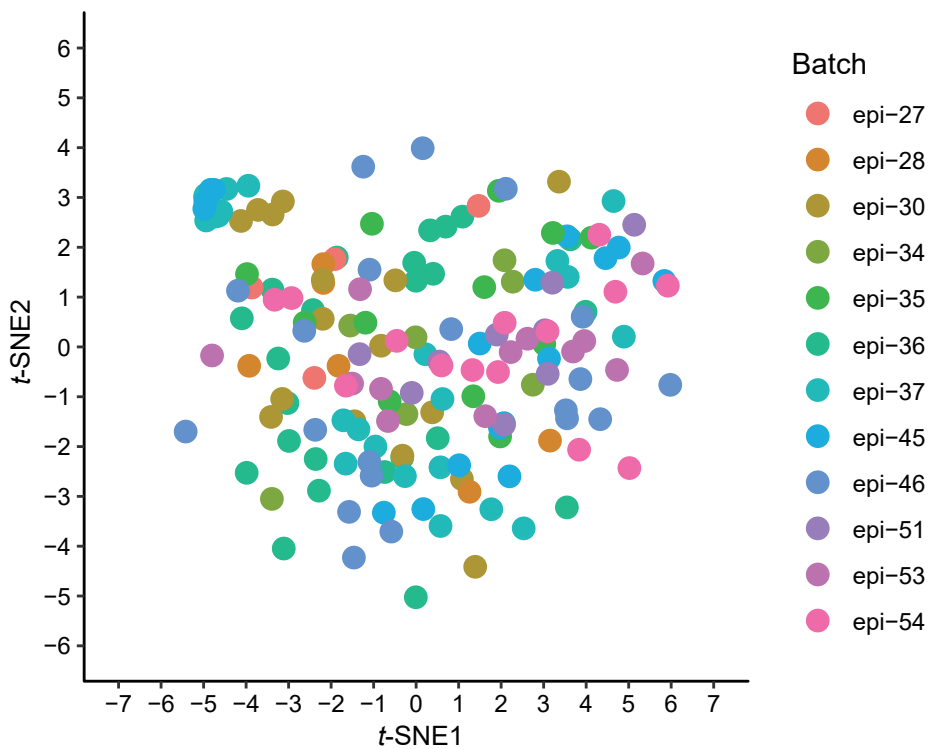**B**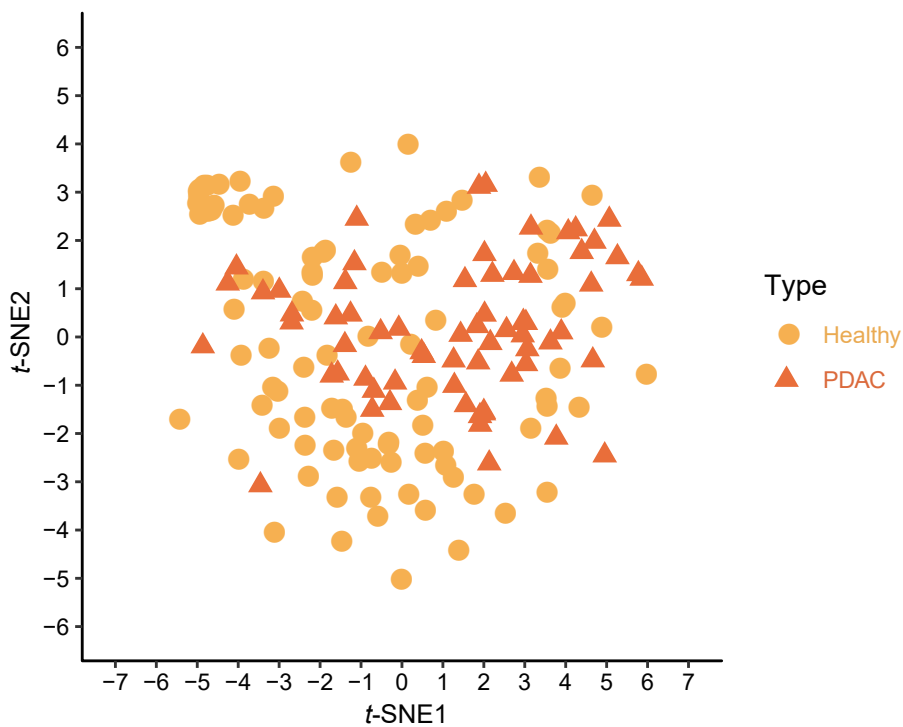

Supplement: Supplementary file 1 — Additional file 1: Supplementary Figure 1. Percentage of reads mapped to the spike-in DNA. A. The 5mC spike-in DNA is specifically enriched in the 5mC libraries. B. The 5hmC spike-in DNA is specifically enriched in the 5hmC libraries. Error bars indicate Standard Deviation (SD). Supplementary Figure 2. Global change in 5mC and 5hmC level in PDAC. A. Boxplot of 5mC peak numbers from healthy controls and PDAC samples shows no significant difference. B. Boxplot of 5hmC peak numbers from healthy controls and PDAC samples shows significant larger numbers of peaks in PDAC samples. C. Genome browser view of the cell-free 5mC distribution in a 5 mb region in chromosome 8. D. Genome browser view of the cell-free 5hmC distribution in a 3 mb region in chromosome 7. The overlapping tracks of healthy and PDAC are shown in line plot. *P < 0.05, **P < 0.01, ***P < 0.001, ****P < 1e−5, Wilcoxon test. PDAC, pancreatic ductal adenocarcinoma. Supplementary Figure 3. Genomic distribution of 5mC and 5hmC peaks. A. 5mC distribution in genomic features. B. Enrichment of 5mC peaks overlapping with distinct genomic elements. C. 5hmC peak distribution in genomic features. D. Enrichment of 5hmC peaks overlapping with distinct genomic elements. PDAC, pancreatic ductal adenocarcinoma; CDS, Coding DNA Sequence; 3′UTR, 3′untranslated region; 5′UTR, 5′untranslated region. Supplementary Figure 4. Comparison of the 5mC and 5hmC peaks. A. Venn diagram of overlap between 5mC and 5hmC peaks. B. Venn diagram of overlap between genes with 5mC modifications and genes with 5hmC modifications. Supplementary Figure 5. GO term enrichment analysis of specifically modified genes. A. 5mC-specific genes. B. 5hmC-specific genes. PDAC, pancreatic ductal adenocarcinoma. Supplementary Figure 6. Genome browser views of examples of specifically modified genes. A. ME1 gene in chromosome 6: 84,095–84,140 kb. B. PACRG gene in chromosome 6: 163,716–163,734 kb. C. FYN gene in chromosome 6: 112,132–112,148 kb. D. RALB gene i [file 13148_2020_898_MOESM1_ESM.zip › Figure S7.pdf]

**A**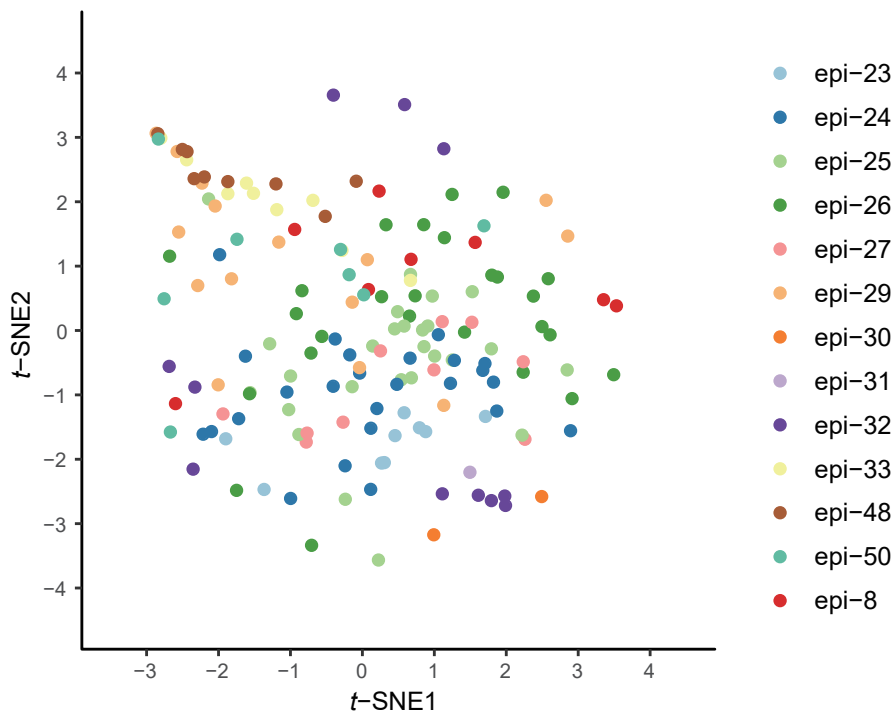**B**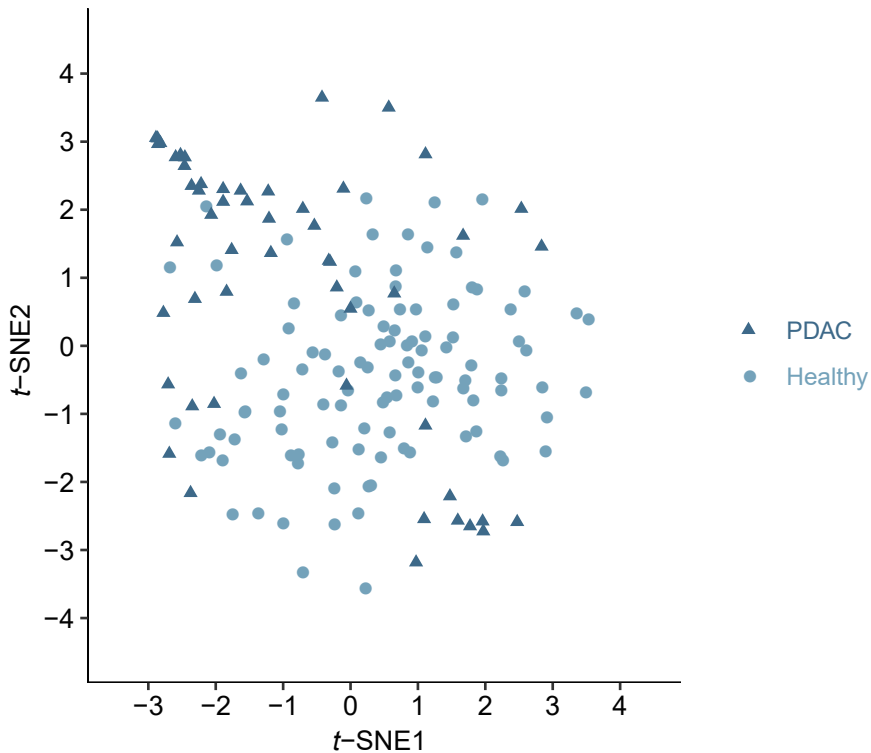

Supplement: Supplementary file 1 — Additional file 1: Supplementary Figure 1. Percentage of reads mapped to the spike-in DNA. A. The 5mC spike-in DNA is specifically enriched in the 5mC libraries. B. The 5hmC spike-in DNA is specifically enriched in the 5hmC libraries. Error bars indicate Standard Deviation (SD). Supplementary Figure 2. Global change in 5mC and 5hmC level in PDAC. A. Boxplot of 5mC peak numbers from healthy controls and PDAC samples shows no significant difference. B. Boxplot of 5hmC peak numbers from healthy controls and PDAC samples shows significant larger numbers of peaks in PDAC samples. C. Genome browser view of the cell-free 5mC distribution in a 5 mb region in chromosome 8. D. Genome browser view of the cell-free 5hmC distribution in a 3 mb region in chromosome 7. The overlapping tracks of healthy and PDAC are shown in line plot. *P < 0.05, **P < 0.01, ***P < 0.001, ****P < 1e−5, Wilcoxon test. PDAC, pancreatic ductal adenocarcinoma. Supplementary Figure 3. Genomic distribution of 5mC and 5hmC peaks. A. 5mC distribution in genomic features. B. Enrichment of 5mC peaks overlapping with distinct genomic elements. C. 5hmC peak distribution in genomic features. D. Enrichment of 5hmC peaks overlapping with distinct genomic elements. PDAC, pancreatic ductal adenocarcinoma; CDS, Coding DNA Sequence; 3′UTR, 3′untranslated region; 5′UTR, 5′untranslated region. Supplementary Figure 4. Comparison of the 5mC and 5hmC peaks. A. Venn diagram of overlap between 5mC and 5hmC peaks. B. Venn diagram of overlap between genes with 5mC modifications and genes with 5hmC modifications. Supplementary Figure 5. GO term enrichment analysis of specifically modified genes. A. 5mC-specific genes. B. 5hmC-specific genes. PDAC, pancreatic ductal adenocarcinoma. Supplementary Figure 6. Genome browser views of examples of specifically modified genes. A. ME1 gene in chromosome 6: 84,095–84,140 kb. B. PACRG gene in chromosome 6: 163,716–163,734 kb. C. FYN gene in chromosome 6: 112,132–112,148 kb. D. RALB gene i [file 13148_2020_898_MOESM1_ESM.zip › Figure S9.pdf]

**A**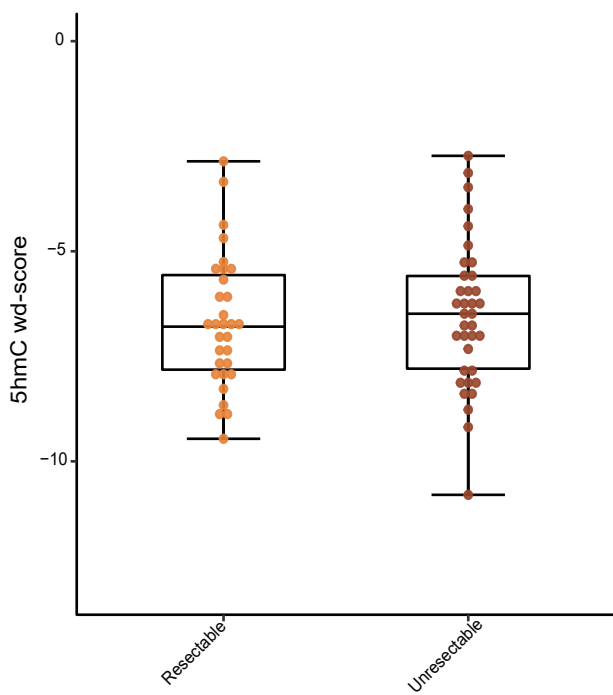**B**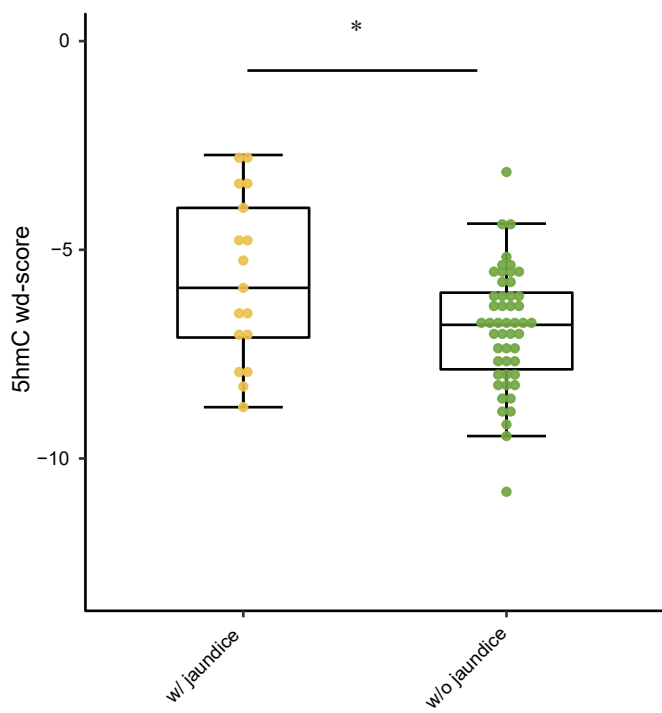

Supplement: Supplementary file 1 — Additional file 1: Supplementary Figure 1. Percentage of reads mapped to the spike-in DNA. A. The 5mC spike-in DNA is specifically enriched in the 5mC libraries. B. The 5hmC spike-in DNA is specifically enriched in the 5hmC libraries. Error bars indicate Standard Deviation (SD). Supplementary Figure 2. Global change in 5mC and 5hmC level in PDAC. A. Boxplot of 5mC peak numbers from healthy controls and PDAC samples shows no significant difference. B. Boxplot of 5hmC peak numbers from healthy controls and PDAC samples shows significant larger numbers of peaks in PDAC samples. C. Genome browser view of the cell-free 5mC distribution in a 5 mb region in chromosome 8. D. Genome browser view of the cell-free 5hmC distribution in a 3 mb region in chromosome 7. The overlapping tracks of healthy and PDAC are shown in line plot. *P < 0.05, **P < 0.01, ***P < 0.001, ****P < 1e−5, Wilcoxon test. PDAC, pancreatic ductal adenocarcinoma. Supplementary Figure 3. Genomic distribution of 5mC and 5hmC peaks. A. 5mC distribution in genomic features. B. Enrichment of 5mC peaks overlapping with distinct genomic elements. C. 5hmC peak distribution in genomic features. D. Enrichment of 5hmC peaks overlapping with distinct genomic elements. PDAC, pancreatic ductal adenocarcinoma; CDS, Coding DNA Sequence; 3′UTR, 3′untranslated region; 5′UTR, 5′untranslated region. Supplementary Figure 4. Comparison of the 5mC and 5hmC peaks. A. Venn diagram of overlap between 5mC and 5hmC peaks. B. Venn diagram of overlap between genes with 5mC modifications and genes with 5hmC modifications. Supplementary Figure 5. GO term enrichment analysis of specifically modified genes. A. 5mC-specific genes. B. 5hmC-specific genes. PDAC, pancreatic ductal adenocarcinoma. Supplementary Figure 6. Genome browser views of examples of specifically modified genes. A. ME1 gene in chromosome 6: 84,095–84,140 kb. B. PACRG gene in chromosome 6: 163,716–163,734 kb. C. FYN gene in chromosome 6: 112,132–112,148 kb. D. RALB gene i [file 13148_2020_898_MOESM1_ESM.zip › Figure S11.pdf]
